# Supplementary figures and images for: Single-cell analysis of the early Drosophila salivary gland reveals that morphogenetic control involves both the induction and exclusion of gene expression programs
Source: PLoS Biol. 2025 Apr 21;23(4):e3003133. doi: 10.1371/journal.pbio.3003133 (PMC12043239; doi:10.1371/journal.pbio.3003133)

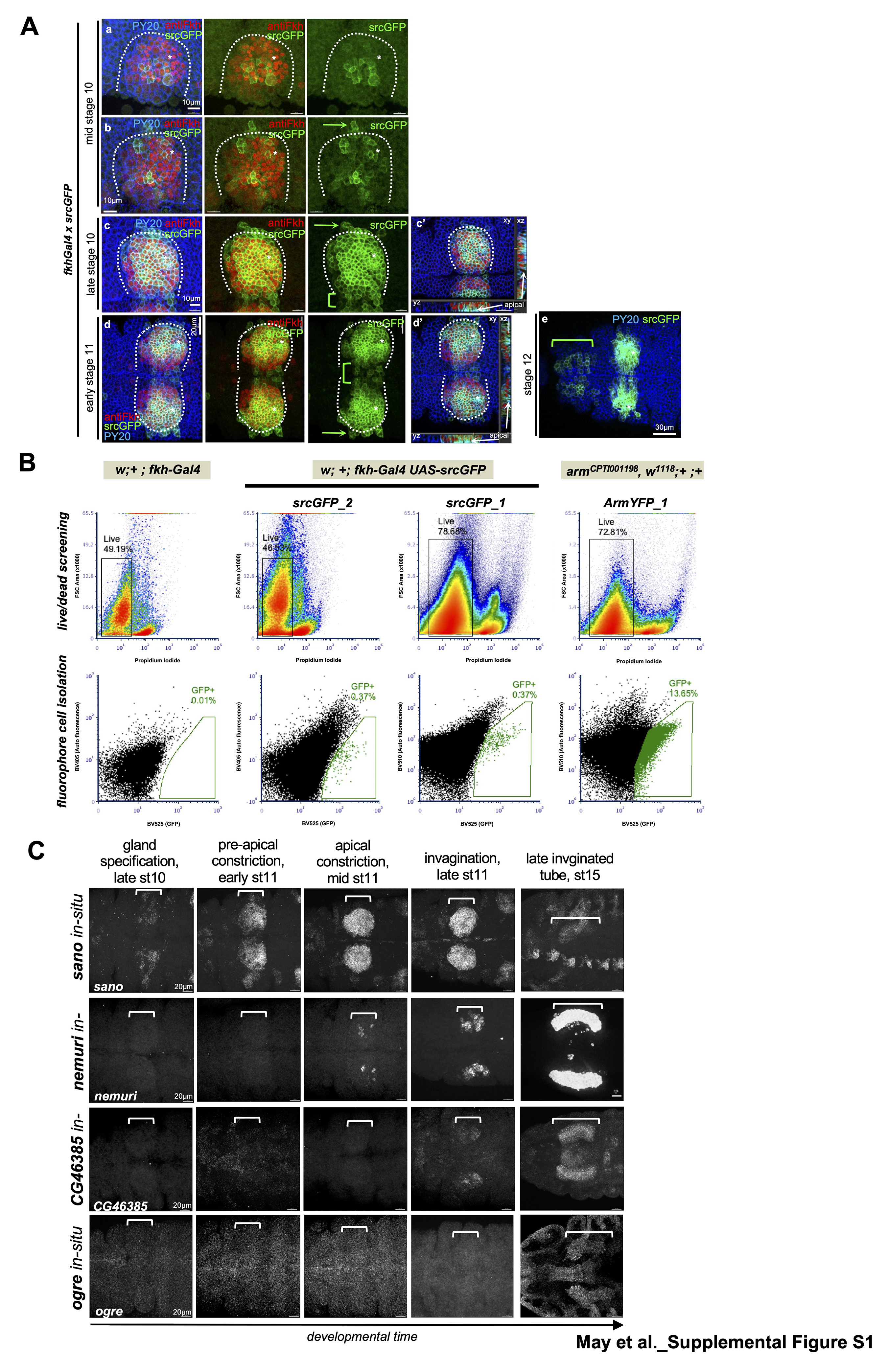

Supplement: S1 Fig — Generation of a single cell transcriptome dataset of salivary gland placodal and epidermal cells. A a, b) At mid stage 10, endogenous Fkh protein, revealed using an antibody against Fkh (red), is already spreading across the placode from the initial expression at the forming pit position (asterisks in all panels mark the position of the future pit). At this stage, srcGFP (green) expressed under fkhGal4 control can be clearly identified to begin to be expressed in central cells of the placode at varying levels. c-d’) At late stage 10 and early stage 11, when endogenous Fkh is seen in all secretory placodal cells (but constriction near the forming invagination point is only just beginning; see cross section panels in c’ and d’), srcGFP expression driven by fkhGal4 is very strong in nearly all placodal cells, with only slightly lower levels in the most anterior cells. Dotted lines mark the boundary of the placode, arrows and green brackets indicate cells outside the placode that express srcGFP when driven by fkhGal4. Cell outlines are marked by an antibody against junctional phosphotyrosine (PY20; blue). B) FACS plots for nonfluorescent control (w; + ;fkhGal4), two srcGFP embryo batches (srcGFP_1 and srcGFP_2; w;fkhGal4 UAS-srcGFP) and one ArmYFP embryo batch (arm[CPTI001198], w[118]; + ;+) used for single cell RNA-sequencing. The top row shows the sorting for live versus dead cells, the bottom row shows the gate for sorting of GFP/YFP-positive cells and the percentage of total cells sorted they comprised. C) Single channels of the HCR in situs in Fig 1F for the indicated genes. (TIF) [file pbio.3003133.s001.tif]

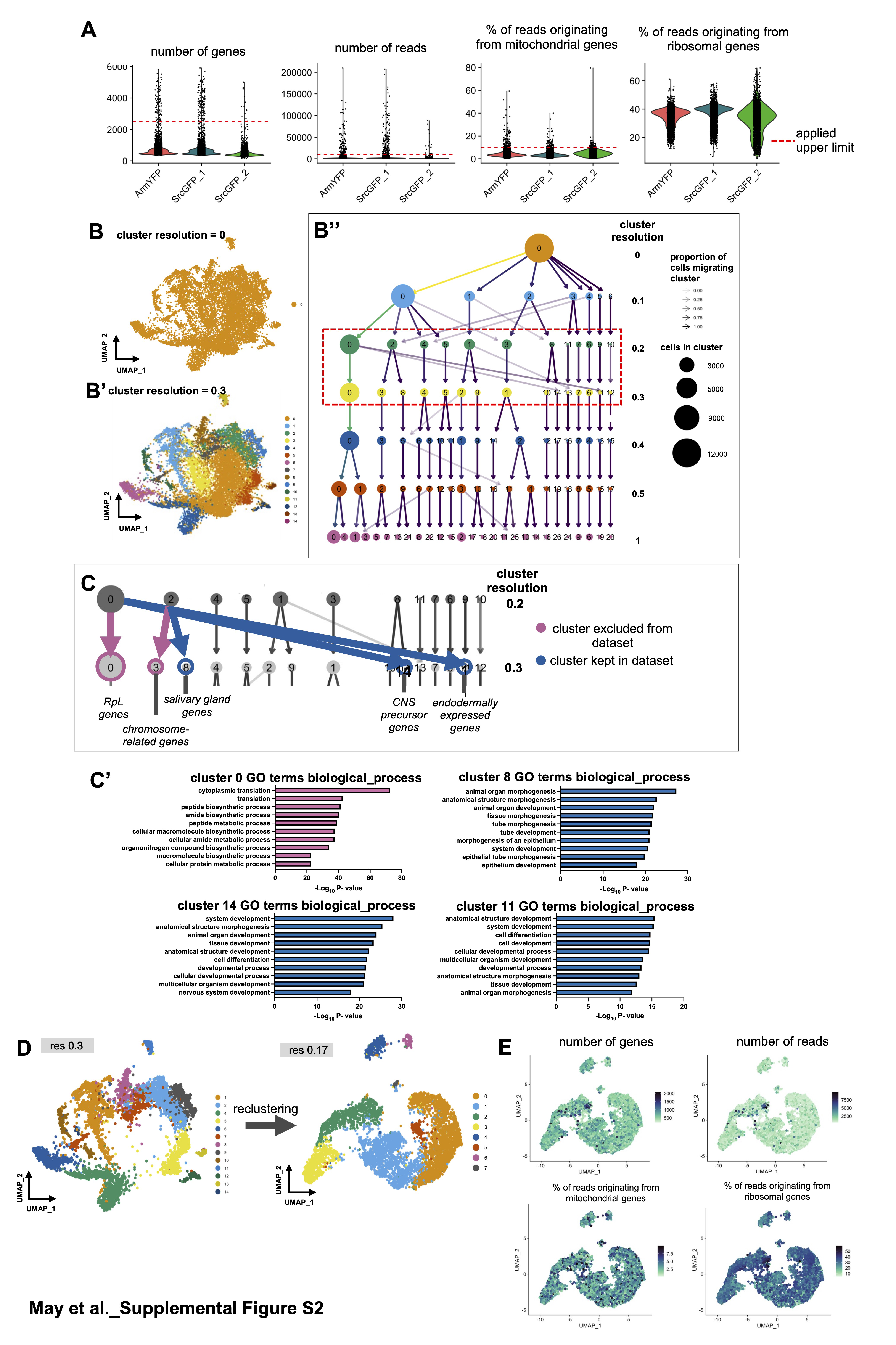

Supplement: S2 Fig — Generation of a single cell transcriptome dataset of salivary gland placodal and epidermal cells. A) Quality control of scRNA-sequencing batches, showing cut offs (red lines) for number of genes, number of reads, % of reads originating from mitochondrial genes and % of reads originating from ribosomal genes. B-B’) UMAP of single cell RNA sequencing at cluster resolution 0 (B) and 0.3 (B’). B’’) Illustrates the emergence and linkage of clusters with increasing resolution generated using the Clustree package in R. Red dotted outline shows clusters assigned at resolutions of 0.2 and 0.3 where further investigations into cluster makers occurred. C) General consensus of markers represented in the clusters emerging at resolution 0.3, purple arrows represent the emergency of cell clusters with markers of low-quality cells and blue arrows represent the emergence of clusters with biologically relevant cell types. C’) Biological process Gene Ontology terms for gene lists generated from resolution 0.3 clusters featured in B, ranked by the -Log10P-value provided by FlyMine curated lists for each ontology term. D) UMAP generated following the reclustering of the original dataset following the exclusion of low-quality cell types identified in C. E) UMAPs displaying the distribution of number of genes per cell, number of reads per cell, % of reads originating from mitochondrial genes and % of reads originating from ribosomal genes to illustrate that these do not cluster in any particular way across the UMAP. (TIF) [file pbio.3003133.s002.tif]

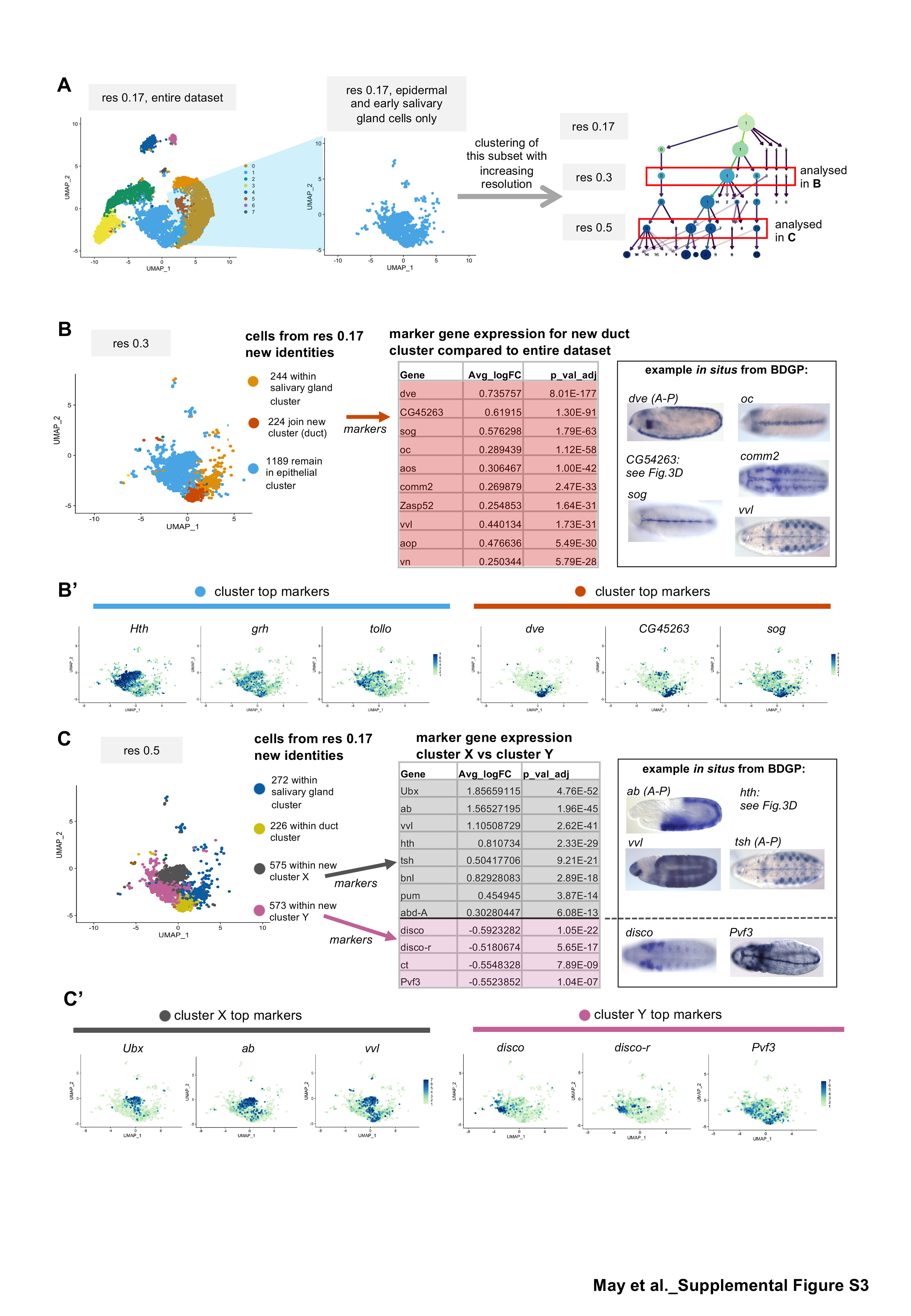

Supplement: S3 Fig — Further analysis of the ‘epidermal and early salivary gland cells’ cluster identified in Fig 2. A) Isolation of the cluster from the resolution 0.17 dataset. The cluster is then further splitting by increasing resolution as illustrated in the flow scheme. B) At resolution 0.3 the cluster splits into three, with 244 cells identified as salivary gland, 244 that form a new cluster, and 1189 that remain in the epithelial cluster. Marker genes and published in situs (BDGP) for the new (red) cluster suggest this to be a duct cluster, as also further analyzed in Fig 3. B’) UMAPs of selected top marker genes for epithelial clusters and new duct cluster identified in B. C) Reclustering at resolution 0.5 splits the epithelial cluster remaining at resolution 0.3 into two further clusters X and Y, and the listed marker genes and published in situs (BDGP) appear to suggest that these clusters could represent more anterior and more posterior epidermis. C’) UMAPs of top marker genes for clusters X and Y identified in C. (TIF) [file pbio.3003133.s003.tif]

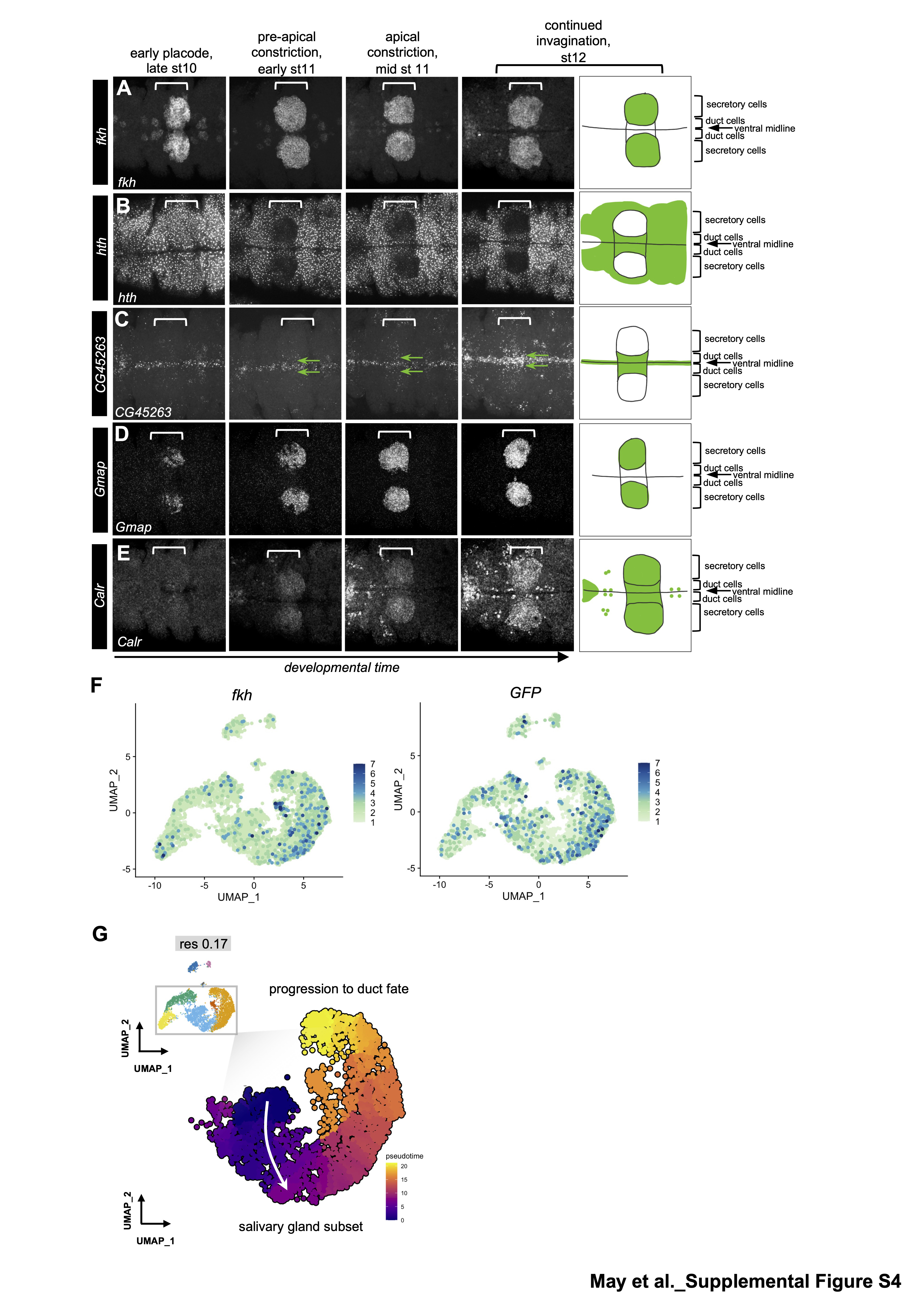

Supplement: S4 Fig — A single cell timeline of mRNA expression changes during salivary gland morphogenesis. A-E) In situ hybridization by HCR of one top marker gene per cluster identified in comparison to fkh expression as shown in Fig 3D, single channels are shown here: hth for the ‘progenitor and early gland’ cluster, CG45,263 for the ‘specified duct cells’ cluster, Gmap for the ‘specified secretory cells’ cluster and Calr for the ‘post specification/late’ cluster. Single in situ channels matching the panels in Fig 3D are shown, with matching schematics explaining the labeling at continued invagination/stage 12. White brackets indicate the position of the salivary gland placodes, scale bars are 30µm. F) UMAP plots based on the clustering in Fig 2A showing expression of fkh and GFP in the combined datasets. G) Pseudotime analysis based on the proposed salivary gland portion of the lower resolution UMAP in Fig 3A. The pseudotime also agrees with a second shorter lineage progression to ductal fate as one possible trajectory. (TIF) [file pbio.3003133.s004.tif]

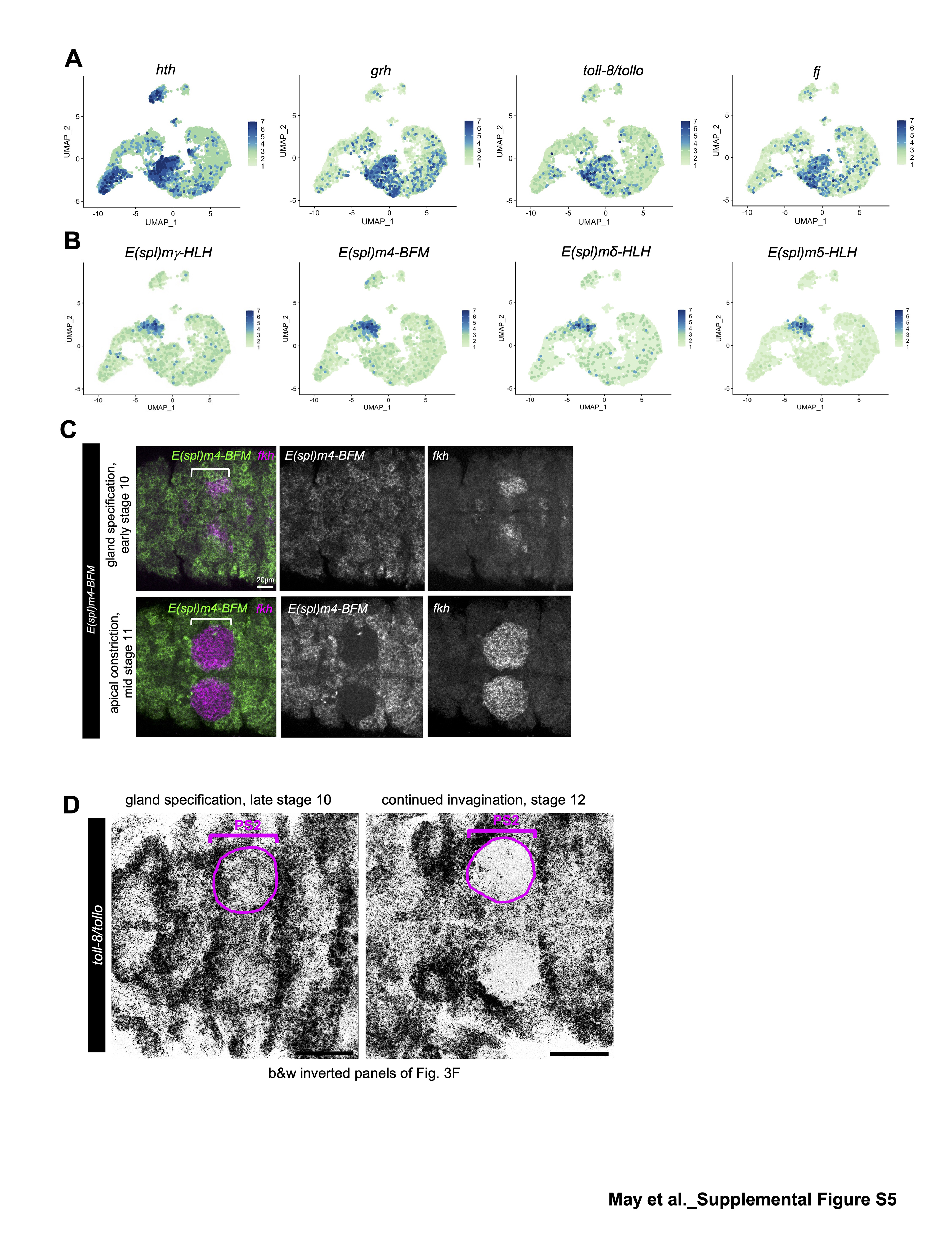

Supplement: S5 Fig — Placode-specific downregulation and exclusion of expression of candidates. A) UMAP plots based on the clustering in Fig 2A showing expression of hth, grh, toll-8/tollo and fj in the combined datasets. B) UMAP plots based on the clustering in Fig 2A showing expression of E(spl)mγ-HLH, BobA and E(spl)m4-BFM in the combined datasets, note the strong increase in expression in the E(sol) cluster compared to the early epidermal cluster. C) Comparison of expression of E(spl)m4-BFM, exemplary for the E(spl) group, between early stage 10 (gland specification) and mid stage 11 (apical constriction), to illustrate the expression in parasegment 2 at the specification stage (fkh expression just initiating) and following downregulation and exclusion of expression in the secretory cells once morphogenesis commences. HCR in situ for E(spl)m4-BFM is shown in green and for fkh in magenta in the overlay, scale bar is 20µm, white brackets indicate the position of the placode. D) Direct comparison of toll-8/tollo in situ by HCR between gland specification stage (late stage 10) and continued invagination stage (stage12) using inverted black and white panels (panels previously shown in Fig 4F), with the position of the secretory cells in parasegment 2 outlined in magenta for one of the two placodes in each panel. (TIF) [file pbio.3003133.s005.tif]

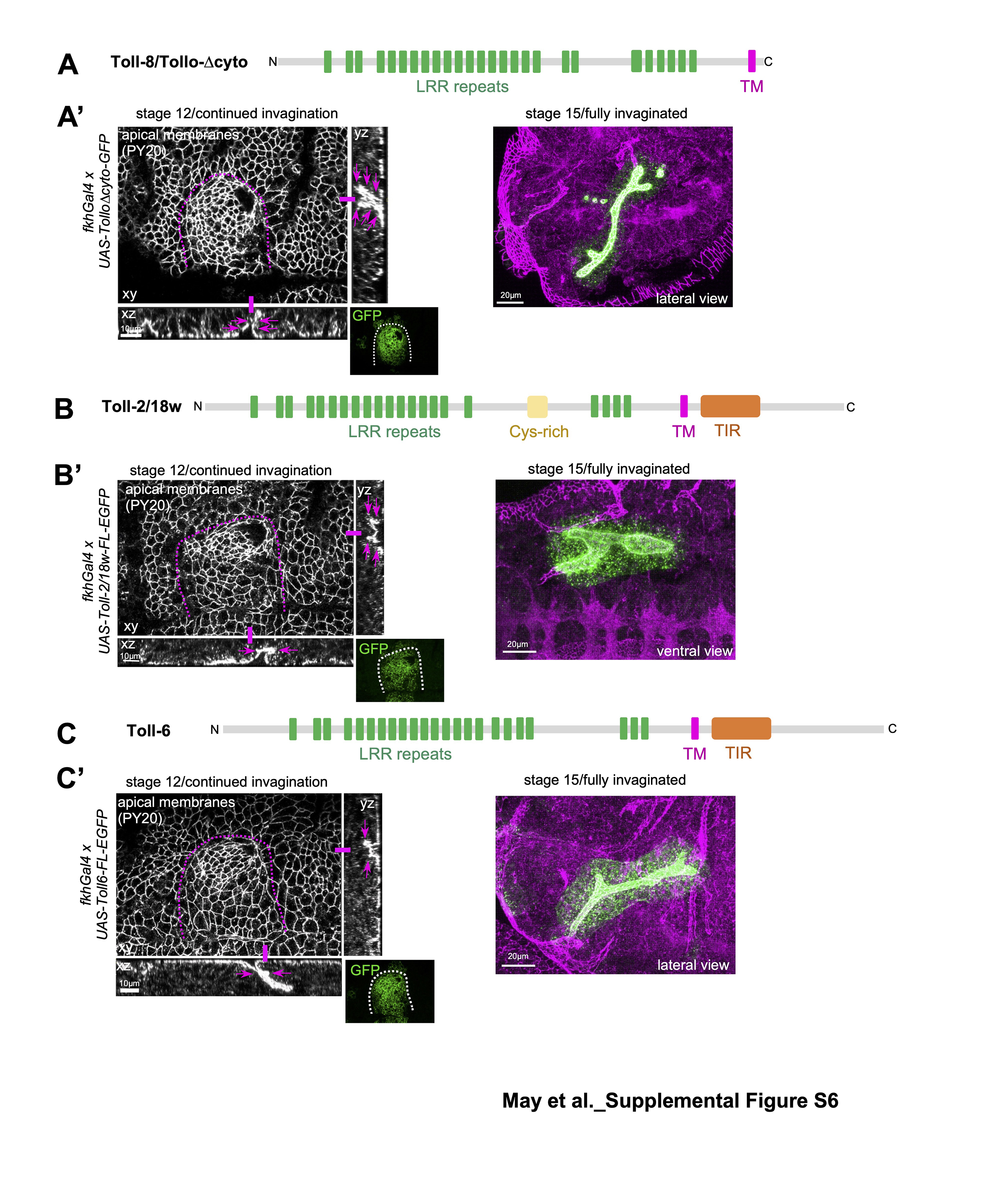

Supplement: S6 Fig — Continued expression of Tollo/Toll-8 disrupts salivary gland tubulogenesis. A, B, C) Schematics of Toll-8/Tollo lacking the intracellular cytoplasmic domain (∆cyto; A), of Toll-2/18w (B) and Toll-6 (C) used for re-expression of in the salivary gland placode using the UAS/Gal4 system. A’, B’, C’) In contrast to control placodes (Fig 5B) where apical constriction begins in the dorsal posterior corner and a narrow lumen single tube invaginates from stage 11 onwards in embryos continuously expressing UAS-Tollo∆cyto-GFP or UAS-Toll-2/18w-FL-EGFP or UAS-Toll-6-FL-EGFP under fkhGal4 control multiple initial invagination sites and lumens form and early invaginated tubes show too wide lumens (magenta arrows in cross-section views). Fully invaginated glands at stage 15 show highly aberrant lumens. Apical membrane are labelled with an antibody against phosphotyrosine (PY20) labeling apical junctions. Dotted lines mark the boundary of the placode, asterisks the wild-type invagination point. Green panels show the expression domain of Tollo∆cyto-GFP, Toll-2/18w-FL-EGFP and Toll-6-FL-EGFP. (TIF) [file pbio.3003133.s006.tif]

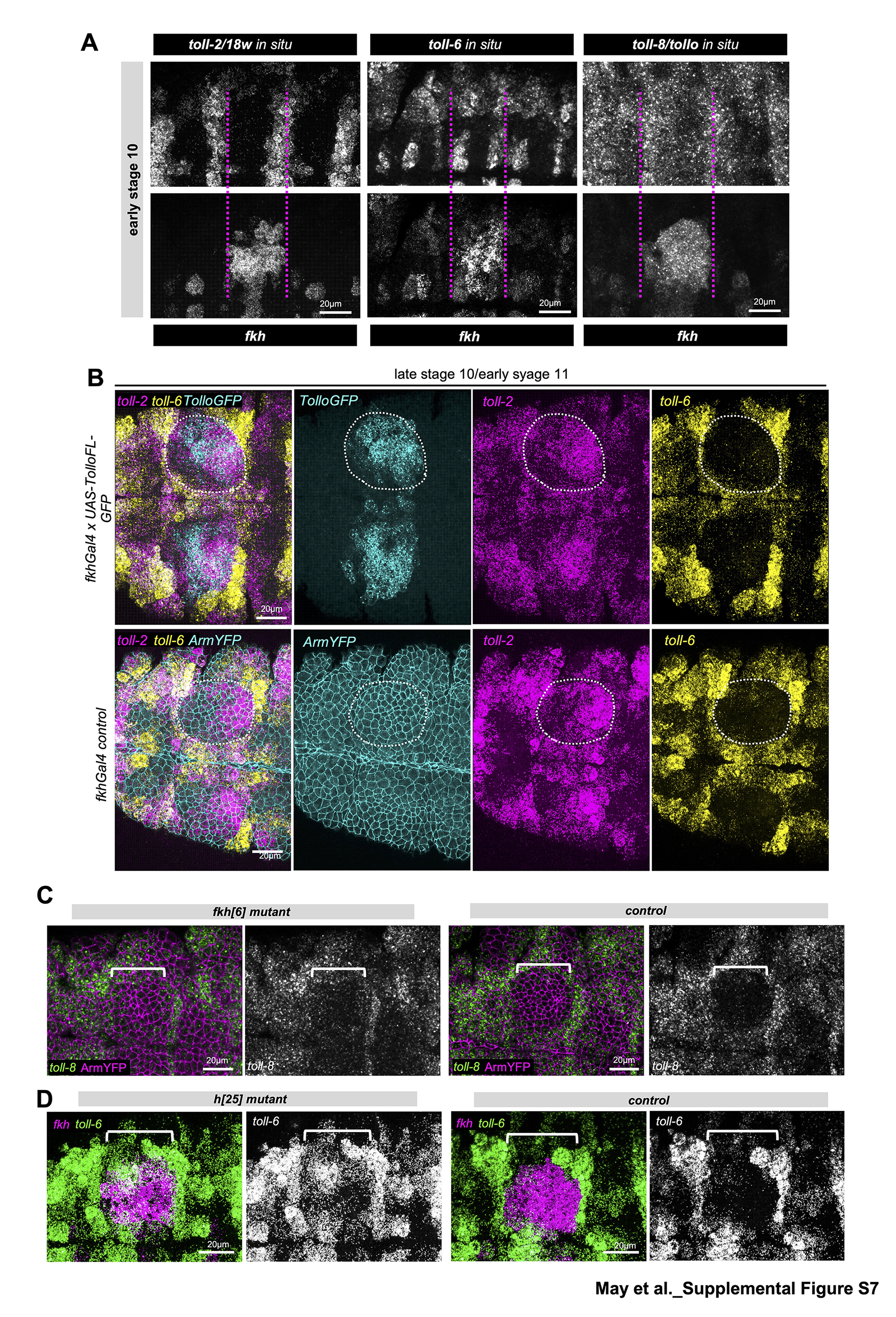

Supplement: S7 Fig — Continued expression of Toll-8/Tollo disrupts an endogenous LRR code required for proper morphogenesis. A) At early stage 10, at the very onset of specification of the salivary gland primordium, toll-2/18w, toll-6 and toll-8/tollo are still expressed in complementary stripe patterns across the epidermis and all overlap with fkh expression. Top row shows in situ hybridizations by HCR for toll-2, toll-6 and toll-8, in comparison to fkh in situ in the lower panels. Magneta lines indicate positions of parasegmental boundaries, thereby illustrating the overalap of all three toll in situ signals with fkh expression at this point. B) Overexpression of Toll-8/Tollo-FL-GFP under fkhGal4 control does not affect either toll-2/18w or toll-6 mRNA levels or localization in the secretory cells of the salivary gland placode. Dotted lines mark the position of the secretory cells for one of the two placodes shown. HCR in situ for toll-2/18w is in magenta, for toll-6 is in yellow, and either the overexpressed Toll-8/Tollo-GFP or ArmYFP to identify the placode position are shown in turquoise; scale bars are 20µm. C) Comparison of toll-8/tolo8 expression analyzed by in situ (HCR) in fkh[6] mutant embryos and control embryos at stage 11. Toll-8/tollo is in green and ArmYFP in magenta. White brackets indicate the position of the placode in parasegment 2, scale bars are 30µm. (TIF) [file pbio.3003133.s007.tif]
