# Supplementary material for: Single-cell analysis of the early Drosophila salivary gland reveals that morphogenetic control involves both the induction and exclusion of gene expression programs
Source: PLoS Biol. 2025 Apr 21;23(4):e3003133. doi: 10.1371/journal.pbio.3003133 (PMC12043239; doi:10.1371/journal.pbio.3003133)
Supplement: Attachment — Submitted filename: Response to reviewers_final.docx [file pbio.3003133.s022.docx]

*We thank the reviewers for their comments and suggestions for improvement. We are pleased that they feel our study and manuscript “represents a technical advance, providing extremely valuable data and information about the transcriptional blueprint of SG specification”, that “the observations with LRR receptors are very interesting”, that this is “this is an interesting study that showcases the utility of scRNA-seq to characterize gene expression in low-abundance cell types such as salivary gland cells” and that the work “will be of significance to the fields of morphogenesis and organogenesis”.*

*We respond in detail to all points raised by the reviewers below.*

**Reviewer #1**:

In this work single-cell RNA sequence analysis is used to explore the morphogenetic events that lead to the formation of salivary glands (SG) in Drosophila, which represent an excellent model system for organ formation. Previous work, including work from the Röper lab, have identified many of the cellular and genetic mechanisms of SG morphogenesis. Here the authors establish an improved protocol to isolate SG cells in a selected temporal window and compare them to otherwise epidermal cells. This approach provides them with enriched populations of selected cells for further analysis. This is an important point as the SG population represents a small proportion of the total amount of cells in the embryo. In fact, some previous sc-RNAseq approaches have failed to clearly identify SG cells. Nevertheless, a recently published study has also succeeded in identifying SG cell types (Peng et al, Dev 2024, doi:10.1242/dev.202097). The authors first provide a comprehensive
description of upregulated genes, particularly in comparison with the rest of the epidermis. Then they evaluate the dynamic progression of expression in the different clusters identified among the SG population, which revealed patterns of activation and cessation of expression that indicated a fine-tuned temporal regulation. HCR-in situ hybridization is used to confirm several identified genes. Finally, the authors identify a group of genes that become excluded from the secretory placode as SG development proceeds in spite of the initial presence there. They focus on the exclusion of Tollo to investigate the functional requirement of this downregulation. They find that the continued presence of Tollo leads to unregulated apical constriction and invagination defects that correlate with aberrant actomyosin accumulation. They propose that Toll exclusion is necessary to avoid interfering with other LRR receptors. 

This work represents a technical advance, providing extremely valuable data and information about the transcriptional blueprint of SG specification. The protocol established, sorting the cells of interest, can be applied to the analysis of different organs and tissues to provide enriched populations for further analysis. However, from the conceptual point of view the advance that this work is providing is much more limited. On the one hand, several of the upregulated identified genes have been already identified, using other classical approaches or by comparable analyses (Peng et al, Dev 2024, doi:10.1242/dev.202097). No functional validation of newly identified factors is presented in this work. On the other hand, the authors highlight the exclusion of specific factors during SG specification. It seems not unusual that besides the upregulation of specific factors, morphogenesis and organogenesis is also linked to the silencing of other factors. Several examples can be found
in the literature (e.g. serpent expression in the endoderm disappears at st 10-11, Rehorn et al 1996; [doi.org/10.1242/dev.122.12.4023](http://doi.org/10.1242/dev.122.12.4023)), and also in the SGs themselves, where it is known that fkh is inhibited in the duct cells (Haberman et al 2003, doi: 10.1016/s0012-1606(03)00140-4) or that ribbon transcriptionally activates and represses different factors (Loganathan et al, 2016; doi:10.1016/j.ydbio.2015.10.016). Therefore, selective exclusion of factors during organ formation does not seem to convey a novel or surprising concept.

*We thank the reviewer for bringing up this point. We respond in detail to the specific questions below, but I would like to comment here that the examples of other ‘excluded’ factors that the reviewer is mentioning are all themselves transcription factors (serpent, fkh) and of course several of the factors we identified and studied are transcription factors or work with these (hth, grh, E(Spl)s). As we discuss in more detail below, what we identified as interesting is that components that we would label as ‘morphogenetic effectors’, i.e. proteins directly impinging on e.g. actomyosin accumulation or activity in time and space, are expressed in all or part of the primordium when specification commences, and their expression then disappears on a very short time scale, whilst remaining in cells just surrounding the salivary gland placode and even in the duct cells of the primordium.*

*In the case of the salivary gland placode this exclusion or directed loss of expression is an important aspect of the tubulogenesis, and the novelty is the level at which the exclusion is implemented, i.e. at the morphogenetic effector rather than TF level.*

At this stage, this work results preliminary and descriptive, as newly identified genes are not functionally tested, and because the analysis of LRR receptors needs to be further investigated (see below). 

Specific comments

1) A recent paper analyzed the organogenetic transcriptomes of Drosophila embryos at different developmental stages (one of them corresponding to the stages analyzed in this work) by sc-RNA seq, with a particular focus on SG. In this published work whole embryos were used as the sample. It would be interesting to compare the results that the authors obtained with those published, to evaluate the advantages of cell sorting and the reproducibility of results upon different experimental treatments.

*As suggested by the reviewer, we have comparatively analysed our dataset and the salivary gland-related dataset provided by Peng et al., 2024, as well as the salivary-gland specific data obtained from Calderon et al., 2022 and now discuss our findings in the discussion of the manuscript, supplemented with comparative tables. We did not include further datasets (such as Seroka et al.,2022) as these did not analyse the early embryonic stages we were interested in.*

*Our comparison revealed that (1) our FACS-enrichment approach allowed us to isolate significantly more salivary gland cells than both other datasets, that (2) our analysis captured many more differentially expressed genes (as defined by both our and also Peng et al.’s approaches), that (3) we identified many genes previously found to be expressed by in situ (BDGP database) in the salivary glands, but even more that this resource had not identified as salivary gland. Although the total number of genes as a reflection of sequencing depth identified by Peng et al. is larger, many of these were only identified in their dataset and are not reflected in the number of differentially expressed genes and therefore might not be salivary gland specific. The high number of differentially expressed genes identified in this study, combined with the larger number of cells and clusters likely representing developmental timelines we feel sets our study apart and makes it valuable resource for the community.*

Table 1. Comparison between single cell sequencing datasets identifying early salivary gland cells.

|  | this study  (GEO: GSE271294) | | | | | | Peng et al., 2024  (GEO: GSE234602) | Calderon  et al., 2022 (GEO: GSE190147, NNv2, pip > 4) |
| --- | --- | --- | --- | --- | --- | --- | --- | --- |
| Resolution | N/A | 0.17 | 0.3 | | | | as GEO | as file* |
| Cluster name | **SrcGFP vs ArmYFP** | **salivary gland** | **early progenitor/ epidermal** | **specified duct** | **specified secretory cells** | **post speci-fication/late SG** | **salivary gland**  **manually labelled cluster** | **salivary gland** |
| Dataset/stage | Stage 10-12 | | | | | | Stage 10-12 | 6-8 hours |
| Number of cells | 3452 vs 2527 | 2127 | 1189 | 224 | 1800 | 653 | 114 | 478 |
| Percentage of dataset | 57.7% SrcGFP | 35.5% | 19.8% | 3.7% | 30.1% | 10.9% | 0.55% | 0.62% |
| Number of genes expressed:  *Findmarkers +*  *(>0 log2fold change in cluster compared to dataset, 10% cells in cluster)* | 642 | 659 | 676 | 955 | 731 | 598 | 2092 | 392 |
| Number of genes differentially expressed, using the limit of Peng et al.:  *Findmarkers +*  *(>0 log2fold change, <0.05 adjusted p-value, gene x expressed in 10% of cells in cluster)* | 346 | 247 | 242 | 77 | 228 | 125 | 157 | 70 |
| Number of genes differentially expressed using the limit of this study:  *Findmarkers +*  *(>0.25 log2fold change, <10E-25*  *adjusted p-value)* | 84 | 53 | 62 | 12 | 43 | 9 | 32 | 12 |

*Calderon et al /content/members/DEAP_website/public/RNA/update/seurat_objects/pred_windows/NNv2/6_8_finished_processing.Rds

Genes expressed in salivary gland (normalising gene names over datasets via FlybaseID validator):

| **Genes expressed in salivary gland** | **This study (0.17 res SG)** | **Peng et al. (SG)** | **Calderon et al. (SG)** |
| --- | --- | --- | --- |
| Total genes | 659 | 2092 | 392 |
| Shared with this study only | 140 | 348 | 38 |
| Shared with Peng et al. only | 347 | 1546 | 65 |
| Shared with Calderon et al. only | 38 | 65 | 156 |
| Shared between all three | 133 | | |

Genes differentially expressed in salivary gland (>0 log2foldchange <0.05 p-value adjusted):

|  | **This study (0.17 res)** | **Peng et al.** | **Calderon et al.** |
| --- | --- | --- | --- |
| Total genes | 247 | 157 | 70 |
| Shared with May Only | 143 | 67 | 16 |
| Shared with Peng Only | 66 | 48 | 7 |
| Shared with Calderon Only | 16 | 7 | 25 |
| Shared between all three | 22 | | |

Genes differentially expressed in salivary gland (>0 log2foldchange <0.05 p-value adjusted) in May et al and the BDGP list of salivary gland expressed genes filtered for “embryonic salivary gland, embryonic salivary gland body, embryonic salivary gland common duct, embryonic salivary gland duct”.

|  | **This study (0.17 res)** | **BDGP salivary gland expression** |
| --- | --- | --- |
| Total genes | 247 | 300 |
| Unique | 166 | 219 |
| Shared | 80 | |

2) Expression of CG 45263 in duct cells in Figs 3 and S3 is unclear. The authors should point to the expression pattern or use magnifications. It is also unclear whether the fkh-Gal4 used to isolate cells is expressed in the duct primordium.

*We are sorry that this was not clear in the original figure. In order to make the establishment and location of future secretory and duct cells clearer, we added schematic panels to Figure 1 and have also explained this in more depth in the manuscript.*

*Furthermore we have added the explanatory schematics shown below to Supplemental Figure S3.*

*We hope this makes the interpretation of expression patterns clearer.*

*With regards to expression of fkh:*

*Endogenous fkh is initially expressed in the dorsal-posterior cells of the salivary gland placode before spreading radially out across the placode in its expression. Only then does fkh expression in duct cells become downregulated downstream of an EGF signal emanating from the ventral midline (Kuo, Y. M. et al. Salivary duct determination in Drosophila: roles of the EGF receptor signalling pathway and the transcription factors fork head and trachealess. Development* ***122****, 1909–1917 (1996); Sánchez-Corrales, Y. E., Blanchard, G. B. & Röper, K. Correct regionalization of a tissue primordium is essential for coordinated morphogenesis. Elife* ***10****, e72369 (2021)).*

*Our isolation of placodal cells is based on the expression of a membrane-tethered GFP (srcGFP; Maybeck, V. & Röper, K. A targeted gain-of-function screen identifies genes affecting salivary gland morphogenesis/tubulogenesis in Drosophila. Genetics* ***181****, 543–565 (2009)) under control of fkhGal4 that is based on a 1000bp fragment of the fkh promoter that drives salivary gland-specific expression (Henderson, K. D., and D. J. Andrew, 2000 Regulation and function of Scr, exd, and hth in the Drosophila salivary gland. Dev. Biol. 217: 362–374; Zhou, B., A. Bagri and S. K. Beckendorf, 2001 Salivary gland determination in Drosophila: a salivary-speciﬁc, fork head enhancer integrates spatial pattern and allows fork head autoregulation. Dev. Biol. 237: 54–67). FkhGal4-driven expression is of course only occurring with a delay compared to endogenous fkh. To illustrate where and when fkhGal4-driven srcGFP is expressed, and in comparison to endogenous Fkh protein, we have added new panels to Supplemental Figure S1, also shown below. The panels also illustrate some cells outside the salivary gland placode where fkhGal4-driven srcGFP is expressed where endogenous Fkh is not present (arrows), potentially accounting for some of the FACS-isolated cells that are not salivary gland.*

3) The observations with LRR receptors are very interesting. A more detailed analysis would be needed to strengthen the conclusions drawn. Actually, it would be very interesting if the authors could test some of the speculations that they put forward in the discussion, as several tools seem to be available.

-It is proposed that Tollo is excluded from the SG placode to prevent undesirable interactions with other LRR receptors (possibly through heterophilic interactions). They find accumulation of 18w at the invagination point, suggesting a role of this receptor in the process. Is 18w required for invagination? Does it modulate actomyosin accumulation in the placode?

*As we discuss and cite in the manuscript, the Beckendorf lab previously analysed Toll-2/18w (Kolesnikov, T. & Beckendorf, S. K. 18 wheeler regulates apical constriction of salivary gland cells via the Rho-GTPase-signaling pathway. Dev Biol* ***307****, 53–61 (2007)). In this study, the authors show that 18w expression initiates at the position of the future invagination pit and then radially spreads out across the placode, as we observe as well and show for the early stages in Figure 6. Their study further showed that 18w-/- salivary glands are misshapen, a phenotype very reminiscent of the gland shapes we observe under Toll-8/Tollo-overexpression. Furthermore, Toll-2/18w overexpression across the placode (they show this, though, using sca-Gal4) also leads to misshapen glands. We have repeated this Toll-2/18w overexpression experiment under the control of fkhGal4 and observe a similar phenotype, and we also observe a comparable phenotype when Toll-6 is overexpressed under fkhGal4 control (now in Suppl. Fig. S6).*

*Our interpretation of these results is that the wild-type gradually expanding expression of Toll-2/18w is key to wild-type ordered cell internalisation of placodal cells, likely through effects that Tolls/LRRs and therefore Toll-2 can exert on apical actomyosin accumulation and function. Loss of 18w perturbs this, as does overexpression of 18w that homogenises any graded expression that is required for wild-type glands (as shown in Kolesnikov, T. & Beckendorf, S. K. 18 wheeler regulates apical constriction of salivary gland cells via the Rho-GTPase-signaling pathway. Dev Biol* ***307****, 53–61 (2007)). Overexpression of Toll-8/Tollo or Toll-6 also interferes with Toll-2/18w endogenous functions (our results and interpretation), because these Tolls can bind to and therefore interfere with endogenous Toll-2/18w. The Zallen lab have previously shown that these three Tolls (-2, -6, -8) can all interact in S2 cell aggregation assays (Paré et al., 2024). Furthermore, the overexpression of Toll-8∆cyto has the same effect as full-length overexpression, supporting that the extracellular domain of Toll-8 and its likely interactions (with Toll-2) are causing the overexpression phenotype, not any effect downstream of the intracellular domain.*

-The results presented are based on the expression pattern of three Toll receptors (18w, Toll-6 and Tollo), and the phenotype of Tollo overexpression. However, it is not addressed whether the defects of overexpression are due to a specific effect of Tollo or just unspecific effects due to the high overexpression.
In this line it would be interesting to see whether the overexpression of the two other Toll receptors produce similar effects.

*Please see the discussion and interpretation above.*

-It would be interesting to understand whether Toll receptors are required and interchangeable for SG development. Thus, could Tollo or Toll-6 (also excluded from the SG placode) rescue the defects of 18w?

*We agree with the reviewer that these would be desirable experiments, but based on the tools available are unlikely to work for technical reasons. Rescue experiments based on Gal4-driven overexpression are notoriously difficult to interpret. The expression level/pattern never matches the endogenous one, and so a failure to rescue cannot be interpreted. As we discuss already above, Toll-2/18w overexpression has been shown by the Beckendorf lab to lead to a salivary gland phenotype, strongly suggesting that the balance in level of a rescue might be tight. Furthermore, our own and the Beckendorf lab’s study show that Toll-2/18w shows a radially expanding expression pattern that likely is key to its function and that we will not be able to replicate using UAS/Gal4, making the possibility of an interpretable rescue very unlikely.*

-It is unclear in the results section the logics of using the Tollo∆cyto construct.

*We hope that our explanations above have illustrated the logic.*

*We have now rewritten the section discussing this in the manuscript, so we hope our logic is now clear.*

**Reviewer #2:** 
In the study by May & Röper, the authors use single-cell RNA-sequencing (scRNA-seq) to characterize gene expression important for salivary gland development. The salivary gland develops from simple epithelial placodes that are specified by homeotic factors Scr/Hth/Exd. To obtain sufficient quantities of salivary gland precursor cells, the authors use a cell sorting approach based on the expression of GFP in these cells driven by fkh-Gal4 using the GAL4/UAS system. In addition to salivary gland cells (orange cluster Fig 2A), this sorting approach also enriched for other cell types including CNS, muscle, hemocyte, anterior midgut, and non-salivary gland epidermal cells (i.e. other clusters, Figure 2A). Using a combination of hybridization chain reaction (HCR) probes and in situ, the authors assay representative marker genes (i) to provide supporting evidence for identity of cells within clusters and (ii) to make a case for dynamic gene expression associated with salivary gland
development. The interpretation of subclusters in terms of dynamics is a reasonable idea, and partially supported by their trajectory analysis. However, the reviewer is concerned that the authors are likely oversimplifying. As cell sorting/fkh-Gal4 likely identified additional cell type, beside the placode cells, how sure are the authors that cells used for the trajectory analysis relate to salivary gland development. In particular, the "early" salivary gland precursor cells (as defined in Fig 3) were first characterized as "non-salivary gland epithelial cells" (Fig 2).

*The clusters identified and named in Figure 2 were determined in their identity based on our analysis of marker gene expression pattern as determined by HCR in situ shown in the figure. A subset was then further analysed in Figure 3 that likely encompassed stages of early salivary gland morphogenesis. In Figure 3, the splitting into subclusters of ‘early placode’, ’specified duct’, ‘specified secretory’ and ‘post specification’ is based again on the HCR analysis presented in this figure across the early timeline of morphogenesis (Fig. 3D). This HCR RNA localisation analysis across early stages of tube formation clearly identified the four clusters as matching the descriptive terms given by us.*

*We agree that the naming of the ‘light blue’ cluster in Figure 2 was confusing and have now renamed this cluster as ‘epidermal and early salivary gland’ as it is likely to contain both ‘epidermal cells’, ‘epidermal cells about to be fated as salivary gland’ and ‘early just specified salivary gland cells’, as their expression profiles are likely to be similar. And to illustrate this, we have further analysed this cluster, as now shown in Supplemental Figure S3.*

*In our analysis in Suppl. Fig. S3 we isolated the blue (‘epidermal and early salivary gland cells’) cluster defined at 0.17 resolution and increased the resolution to 0.3 and 0.5 and analysed the resulting new clusters for marker genes to define better the cell types combined in the original blue cluster.*

*This revealed that at resolution 0.3, a cluster (orange) emerged that was clearly salivary gland (based on marker expression), the red cluster emerged as the duct cell cluster also identified in Figure 3A, and we show the complete list of differentially expressed marker genes for this duct cluster in Suppl. Fig. S3B and Supplemental Table S7, as well as examples of BDGP database in situ images of several of these genes.*

*When resolution was increased further to 0.5, the remaining blue epithelial cluster split into two new clusters (Suppl. Fig. S3C, clusters X and Y), that appear to mark more posterior (X) versus more anterior (Y) epidermis, as judged by BDPG in situs.*

*See also our explanation with regards to a related question raised by reviewer 4 below.*

Attention should be made to clarify where and when the fkh-Gal4 driver supports expression, as it is likely expressed in a broader area (perhaps early at least) and perdurance of the GFP could mean that cells besides salivary gland precursors were isolated by FACS and present with the dataset. They recognize this is the case, as CNS, muscle and hemocyte cells were found. However, the relationship of the large clusters - salivary gland, non-salivary gland, and enhancer of split (Fig 2A, orange, light blue, green) should be more closely dissected. If fkh-Gal4 driver is as localized to the salivary gland as shown in Fig 1A, it is unclear how cells other than salivary secretory cells were identified; nevertheless, they were. Is the fkh-Gal4>GFP nonsensitive, or could other cells have arisen in the dataset due to cell doublets? Furthermore, it should be determined whether fkh-Gal4 is able to identify cells as the authors suggest (i.e. stage 10 doesn't show staining in Fig 1A).

*Please see above in our response to reviewer 1 details about the fkhGal-driven expression of UAS-srcGFP (membrane-tethered GFP) that we used for the cell isolation by FACS and see the detailed analysis of the expression pattern that we now include. FkhGal4-driven srcGFP is present in stage 10 placodes, but we originally displayed the panels in Fig.1 A with identical settings, leading to the already present placode-specific GFP not very visible compared to the increasingly higher levels later on. We now show the first panel with a longer exposure, so the specific GFP in placodal cells is already visible here (as it is in the new panels in Supplemental Figure S1) and we explain this in the legend.*

*We now show further quality control data and cut offs for analysis that were used to ensure that doublets were eliminated from the analysis (Suppl. Fig. S2A).*

*During the quality control stage, cells with more than 10,000 reads were excluded from analysis, this limit was applied to remove the majority of doublets from the dataset, this is now documented in the now publicly available github and in the Methods section of the paper. We now show further quality control data and cut offs for the removal of low quality cells from the dataset in Suppl. Fig S2. The clusters within the finalised dataset we show do not cluster by reads or number of genes per cell so, we are confident that the other cell types within the dataset have not arisen as a result of doublets.*

In summary, this is an interesting study that showcases the utility of scRNA-seq to characterize gene expression in low-abundance cell types such as salivary gland cells (representing 0.4% of the total). While the authors recognize that their sample is temporally diverse (stages 10-12), they need to examine whether the different clusters identified (Fig 2A) relate to time or space/cell types. 

In particular, the authors should explore the possibility that the "non-salivary gland epidermal cells" (Fig 2A) are progenitors also for the enhancer of split enriched cells. If that is the case, Tollo may simply be associated with the designation of two distinct cell types (retained in Espl cell cluster but lost in salivary gland). The message may stay the same, but it seems like an oversimplification as presented to focus on the exclusion of Tollo as an important finding.

The text is clear and the approach is logical. Below are points/questions that if addressed could help to make this interesting study stronger.

Major comments:

1) The focus on Tollo exclusion does not seem well supported. The data shown in Fig 4E is not convincing; and while the results shown in Fig 6A,B are better - this effect needs to be quantified and examined more closely. For instance, if tollo/toll-8 is not excluded from the salivary gland placode then is toll-2 expression affected? Is the toll-2 expression lost? In Fig 6B, tollo expression appears to be retained at the bottom of the placode, whereas toll-2 emerges at the top.

*To demonstrate the downregulation and exclusion of toll-8/tollo expression more clearly, we have now quantified it. We have used late stage 10 embryos (gland specification) and embryos at late stage11 to stage 12 (continued invagination) and have measured toll-8/tollo HCR fluorescence intensity per area for the area of the secretory salivary gland placodal cells in parasegment 2 (as identified by fkh expression) and have compared this in each embryo to toll-8/tollo HCR fluorescence intensity per area for the an equivalent patch of cells in parasegment 1, where the overall intensity does not change between these stages. Expressing toll-8/tollo intensity as a ratio between PS1/PS2, as this allows to compare between different embryos with variation in HCR fluorescence signal, this quantification revealed a clear loss of fluorescence intensity for HCR for toll-8/tollo in PS2 between late stage 10 and stage 12 (now Fig. 4F’).*

*We also now show the earliest and second-to-last panel of Fig. 3F for toll-8/tollo expression in Suppl. Fig. S5C as inverted b&w panels, with the position of the placode and parasegment 2 labelled, as this clearly shows the loss of HCR label.*

*Furthermore, with regards to potential inter-regulation of Toll expression, we have now analysed toll-2/18w expression and also toll-6 expression when Toll-8/Tollo is overexpressed and find that there is no change to either of the other two Tolls at the transcriptional level (see Suppl. Fig. S6).*

*This is in agreement with the fact that our analysis of overexpression of full-length versus ∆cyto versions of Toll-8/Tollo has demonstrated that the effect, as already discussed above for reviewer 1, is dependent on the extracellular domain and not the intracellular tail. This suggests strongly that it is likely the ability of Toll-8 to bind to and therefore interfere with other plasma-membrane-localised Tolls/LRRs that is mediating the effect on the morphogenesis that we see. The cytoplasmic domain of Toll-8 is not required for the overexpression phenotype, therefore making direct intracellular effects of excessToll-8 (e.g. on transcription, translation etc.) very unlikely.*

*With regards to the pattern of expression and exclusion of Tolls:*

*The endogenous expression of toll-8/tollo is downregulated/excluded within the forming secretory cells of the salivary gland placode, not the future duct cells (the two groups of cells referred to by the reviewer as top and bottom of placode, see further explanations for this distinction above in our response for reviewer 1). We have now included explanatory schematics illustrating the transcriptional control of future secretory and duct cells within the placode in Figure 1 (see above) and have also included explanatory schematics in Suppl. Fig. S4.*

*We also now show the panels shown as two-channel images in Fig. 6A as separate channels in Suppl. Fig S 7A so that the overlap of the initial expression of fkh and of toll-2, toll-6 and toll-8 at this early stage is clear.*

2) Furthermore, the finding that Scr mutants eliminate tollo-/toll-8 exclusion doesn't provide much insight as the tissue is likely transformed and no longer able to form a salivary gland placode. If the placode forms, then the author should provide supporting evidence.

*Several papers stretching back more than 20 years by various labs have demonstrated that Scr (in conjunction with Exd and Hth) is key to conferring salivary gland identity to cells in parasegment 2 and is the top most transcription factor in this specification cascade. Without Scr expression, cells retain their epidermal fate, do not take on salivary gland fate, and ectopic expression of Scr leads to the formation of ectopic salivary gland placodes (and tubes) in anterior segments. Analysis of expression of presumed salivary gland-specific factors in scr-/- mutants compared to control has been used repeatedly to demonstrate expression of genes as part of the ‘salivary gland programme’. Please see below in our response to reviewer 4 point 6 for many details and citations in relation to the point raised here.*

3) Would the authors expect that overexpression of any Toll gene would perturb the Toll signaling code/morphogenesis of the salivary gland? And if that is the case, are the phenotypes arising from ectopic expression of Tollo that surprising? The reason this is important, in my opinion, is because Tollo may simply be a gene that is associated with development of "non-salivary gland epidermal cells". Is it really surprising that as cells within the placode develop -  some become salivary gland duct cells, others become secretory cells, and yet others specify epidermal cells?

*We think it is very unlikely that Tollo/Toll-8 is involved in fate specification in the salivary gland placode. It is well established how the differentiation of placodal cells into duct and secretory cells is achieved (EGF emanating from the ventral midline, as explained above and also in the manuscript in detail) and also what the phenotypes are when this differentiation balance is affected, and they are very distinct to what is observed here (we have previously analysed this in detail in another study; Maybeck, V. & Röper, K. A targeted gain-of-function screen identifies genes affecting salivary gland morphogenesis/tubulogenesis in Drosophila. Genetics* ***181****, 543–565 (2009)).*

*We also present data in the manuscript, including some that were included in the original version, that argue against this:*

*🡪 the overexpression phenotype is also observed when UAS-Tollo∆cyto is expressed, thus there cannot be a signal at least from the overexpressed Toll directly to the nucleus*

*🡪 the fact that fkhGal4 remains expressed (for several hours, until the end of morphogenesis, as evidenced by the continuously increasing levels of GFP in embryos expressing e.g. tagged Tollo or Tollo∆cyto) is another indication that fate is not affected (unless perdurance of the expressed protein stretches across all of embryogenesis, we show expressing glands at stage 15).*

*🡪 there is normal CrebA expression (a marker of secretory cells that is upstream of secretory capacity in the glands and downstream of Scr) when Tollo is overexpressed (for the role of CrebA in the salivary glands see: Abrams, E. W. & Andrew, D. J. CrebA regulates secretory activity in the Drosophila salivary gland and epidermis. Development* ***132****, 2743–2758 (2005))*

*🡪 even though the invagination is misshapen under Tollo overexpression, there is still invagination, whereas in Scr-/- mutants where fate is lost, not invagination is observed at all.*

*Please see our reasoning for the cause of the overexpression phenotype in response to questions asked by reviewer 1 above.*

*Figure for reviewer: The above panels show a representative embryo under fkhGal4-driven Toll-8/Tollo-GFP overexpression at stage 11 (invagination). In these overexpressing glands, CrebA, a key marker of secretory fate and capacity of the salivary gland placodal cells with secretory fate, is present as in wild-type gland (see Fig. 4D for comparison; note: the CrebA antibody from the DSHB displays a non-specific punctate background not in the nucleus), demonstrating that with Toll-8/Tollo overexpression the placodal cells have not lost their secretory fate.*

4) The reviewer would caution the authors to explore the relationship between clusters identified in Fig. 2A before focusing then in Fig 3 onwards exclusively only on a subset of the clusters. In particular, the trajectory analysis in Fig 3E suggests that cells previously characterized as the "non-salivary gland epidermal cells" (Fig 2A) may correspond to a progenitor population that relates to salivary gland development as well as to that of the epidermal cells. For instance, in Fig 3E, purple color is found both to the right (closer to salivary gland cells) as well as to the left (closer to the enhancer of split-enriched cells).

*Please see our response above, we have now analysed this cluster in much more detail in new Suppl. Fig. S3.*

*We now show the toll-8/tollo expression UMAP in Suppl. Fig. S5A, illustrating the highest expressed group of cells within the ‘epidermal and early salivary gland cells’ cluster. This cluster, we agree, is likely a combination of epidermal cells about to be fated as salivary gland, of early salivary gland fate cells and cells that will remain epithelial in fate plus some that are or are to become neuroectodermal in fate and that progress to the E(spl)cluster and then neuronal fate. The pseudotime analysis supports this, as a second timeline, now shown in Fig. 4E runs from the E(spl) towards the neuronal cluster, and could originate in the large central ‘epidermal and early salivary gland cells’ cluster.*

5) The authors should add fkh and gfp expression to the dot plot in Fig 3B. Is fkh expressed by the majority of cells in the cluster? gfp would serve as a nice reference control.

*We now show fkh and GFP expression in UMAP plots in Suppl. Fig. S4, which we think is more informative.*

6) The authors should show a feature UMAP plot, displaying individual cells within the UMAP associated with Tollo expression. In particular, Tollo expression in the UMAP of Fig 3A and Fig 2A should be displayed, so that a comparison to  "early salivary gland" and "non-salivary gland epidermal cells" clusters can be made. Again, the reviewer's concern is that Tollo is simply a gene that is expressed in other epithelial cells that will not turn into salivary glands (e.g. Enhancer of split cluster cells?). By incorporating a discussion of the potential alternative trajectory towards whatever fate the Enhancer-of-split cluster cells form, the paper might be clearer. It would not detract from the trajectory analysis of Fig 3E, to label "early" as a progenitor population.

*In our view, and this might not have been clearly enough explained in the original version of the manuscript, the ‘early’ cluster (now labelled ‘epidermal and early salivary gland cells’ in Figure 2A) is likely still a mix of what the reviewer calls a progenitor population combined with cells that are already along the salivary gland developmental trajectory, but in early stages.*

*Our analysis now shown in Suppl. Fig. S5 seems to suggest that cells of this cluster located in the UMAP towards the E(spl)-enriched cluster (new clusters defined as X and Y in this figure in C,C’) are epidermal clusters when looking at marker gene expression between these clusters, they seem to define more anterior versus more posterior epidermis. Nonetheless, in biological terms the E(Spl)-expression has been linked to epidermal development of neuroectodermal cells (Knust et al., 1992, Genetics), some of which will be singled out to become neuronal precursors that become specified around the early stages of salivary gland specification and morphogenesis, and these precursor then delaminate as neuroblasts from the embryonic epidermis in a segmental pattern. Thus, the downregulation and exclusion of expression of E(spl) genes might eliminate the ‘neuroectoderm’ path from placodal cells, as no neuroblasts are specified within the placode. The ‘epidermal and early salivary gland cells’ cluster in Fig. 2A is therefore potentially the starting point for several trajectories, towards salivary gland fate and then secretory phenotype, towards salivary gland fate and then duct phenotype and towards the E(spl) cluster/neuroectodermal fate and then neuronal fate (now shown as a potential pseudo-timeline in the E(spl) cluster analysis in Figure 4).*

*We have also added expression plots based on the UMAP for fkh and GFP to Supplemental Figure S4, and plots for tollo, hth, fj grh and E(spl) genes to Supplemental Figure S5.*

7) With regard to trajectory analysis - does duct form before secretory cells? If not then can the trajectory analysis support the pseudotime model? In Fig 3F, the genes bnl, sano, fkh, fog, Gmap, Fili, nur, ogre, Calr appear to support a temporal progression; however dynamic expression is only shown for Gmap in Fig 3D. In other words, the dynamic expression may pertain to only this particular subset of genes. The other marker genes (Tollo, hth, CG4528) might represent other cell types - not "earlier" salivary gland cells. If this is the case, then the exclusion of Tollo has to be rephrased as a cell fate decision. It would also help to show dynamics associated with genes besides Gmap such at bnl, sano, fog, fili etc.

*Figure 3D illustrates spatio-temporal changes for all genes analysed:*

*- fkh spreads from a dorsal-posterior position radially across the placode (as published previously by us and others)*

*- hth becomes increasingly downregulated/excluded in expression*

*- CG45263 is expressed at the ventral midline, but the signal extends further into the duct cells as time progresses (now illustrated more clearly with schematics in Supplemental Figure S4)*

*-Gmap increases from a ventral patch of secretory cells to the whole secretory part of the placode over time*

*- Calr expression onset is only at a later time point in secretory cells and then increases*

*As already explained above, our data (∆cyto expression as well as retained CrebA labelling) demonstrate that Tollo/Toll8 is highly unlikely functioning as a cell fate switch.*

8) The expression of fkh-Gal4 is shown in Fig 1A using anti-GFP staining is confusing. No expression is detected at stage 10 and yet the authors claim that cells of stage 10 (gland specification?) were identified. How can this be the case? Is the staining lacking sensitivity? And if that is the case, were cells outside the salivary gland placode expressing GFP? Which cells are these?

*The expression was only not very visible in our original version of the figure because all panel images were taken at comparable laser setting to illustrate the increase in expression. We now show the stage 10 panel at increased brightness to illustrate the expression already at this stage. Furthermore we have added a section to Supplemental Figure S1 that clearly shows the pattern of fkhGal4-driven srcGFP expression in comparison to endogenous Fkh protein (see above for reviewer 1).*

9) How were the scRNA-seq data processed to control for doublets? How many cells were eliminated and what steps were involved.

*Please see our response above with regards to doublets and quality control cut offs (shown in Suppl. Fig. S2).*

10) The datasets should be deposited to GEO at the time of review.

*All datasets are available on GEO, though currently still only through private link until publication. Please use the instructions and link for access provided by GEO:*

*‘The following secure token has been created to allow review of record GSE271294 while it remains in private status:*
***cvgtaqsypbcfryx*** *Please note the following points:
- This token allows anonymous, read-only access to GSE271294 and associated accessions while they are private;
- Treat the token as you would a password and realize that the token provides access to GSE271294 to anyone who uses it - we recommend you do not include the token anywhere except in secure email to journal editors;
- If you want to revoke this access token, you can click the 'Reviewer access' button on your record which exposes the option to revoke this token.

Send the following information to journal editors who will circulate to reviewers requiring access to your private data:

----------
To review GEO accession GSE271294:
Go to* [*https://www.ncbi.nlm.nih.gov/geo/query/acc.cgi?acc=GSE271294*](https://www.ncbi.nlm.nih.gov/geo/query/acc.cgi?acc=GSE271294) *Enter token cvgtaqsypbcfryx into the box
----------

Regards,
The GEO Team’*

Minor points:
11) Fig 2C - It's unclear what comparisons were made to calculate the fold change. Is the orange box "early vs early" whereas the blue box is "specified duct vs early" and the green is "specified secretory vs specified duct"? In general, attention should be paid to providing more information in the figures and/or legends.

*I assume the reviewer is referring to our analysis in Figure 3 and in particular 3C.*

*The method used to generate log2fold change values in this paper was through Seurat’s differential expression functions, either ‘findallmarkers’ or ‘findmarkers’ (a general breakdown of these functions can be found under the heading Finding differentially expressed features (cluster biomarkers) in the introductory tutorial for using Seurat;* [*https://satijalab.org/seurat/articles/pbmc3k_tutorial.html*](https://satijalab.org/seurat/articles/pbmc3k_tutorial.html)*).*

*The values of log2fold in the paper are based on the value for each gene in the avg_log2FC column and using the default Wilcoxon Rank Sum test.*

*We have now added a table (Table 2) to the methods, listing for each figure panel the groups that were compared and this is now also generally explained in the figure legends.*

12) Fig 4C - what comparison is made here? E(spl)-enriched cluster relative to what? Early? Is this evidence for two roads that progenitor cells might take… salary gland versus E(spl)-expressing epidermal cells?

*In Figure 4C the enrichment is derived from analysis of the E(spl)-enriched cluster versus the rest of the dataset. As explained above, this is now added to the figure legends, and a table in the methods lists this for all figure panels.*

**Reviewer #3:**

This work by May and Röper uses single-cell RNA sequencing to investigate the genetic regulation of salivary gland (SG) morphogenesis in Drosophila. The authors establish an improved protocol to isolate SG cells during a specific time window, based on comparison with epidermal cells, enriching the desired cell population for analysis. This is crucial as SG cells are a small fraction (less than 1%) of embryonic cells, and past sc-RNAseq methods struggled to identify them. 
The authors describe upregulated SG genes compared to the epidermis and assess dynamic expression changes in SG clusters, revealing tightly regulated temporal patterns of gene expression. HCR in-situ hybridization confirms several identified genes. They identify genes excluded from the secretory placode during SG development, focusing on Tollo's exclusion. Continued Tollo presence leads to unregulated apical constriction and invagination defects, associated with abnormal actomyosin accumulation. The study further concludes that Tollo regulation is downstream of Scr.
Albeit mainly descriptive, this work is important, and the data analysis reveals interesting genetic data. The work gives an overview of the dynamic genetic control of SG morphogenesis and will be of significance to the fields of morphogenesis and organogenesis.

Major comments:

1 - A recent study by sc-RNAseq analysed the transcriptomes of Drosophila embryos at two different stages and succeeded in identifying SG cell types and transcripts (Peng et al., Development 2024, doi:10.1242/dev.202097). These results should be mentioned, and it would be interesting to compare them with the current data. Particularly since the work by May and Roeper seems more extensive and SG specific.

*Please see above in the response for reviewer 1 with regards to the detailed comparative analysis we have undertaken and added to the manuscript.*

2 - Expression of CG45263 is not clear from the panels on figure 3 and figure S3, where the expression seems to be restricted to the nervous system. These panels should be redone to clearly show what the authors describe in the text.

*The images of CG45263 expression as well as of the other genes shown in panel 3D and current Figure S4 are z-projections of the epidermal and salivary gland-fated cells. The HCR labelling for CG45263 is initially within the midline epidermal cells and then spreads into the salivary gland duct-fated cells, this is now explained in greater detail in Figure 1 and is also shown schematically in Figure S4.*

Minor comments:
The authors should explain the rationale for using Tollo without a cytoplasmatic domain (UAS-TolloΔcyto-GFP).

*We are sorry this was not well explained in the original version of the manuscript. Please also see our responses to questions raised by the other reviewers.*

*We now explain the rationale and conclusion from this experiment more clearly in the manuscript.*

**Reviewer #4:**

PBIOLOGY-D-24-01652

In this paper, May and Röper investigated the gene expression profiles in salivary gland and ectodermal cells by performing single-cell RNA sequencing analysis. The authors purified fkh> Gal4-expressing salivary gland cells and whole ectodermal cells (including salivary gland) expressing the pan-ectodermal gene arm using FACS sorting, an approach different from the most of other scRNAseq works analyzing cells from whole embryos. Cell data from the two sources are combined and classified into multiple groups. The authors identified a group of genes with low expression in the salivary gland compared to the ectoderm. One of the genes was tollo/toll8, a leucine-rich repeat membrane protein involved in cell sorting. Forced expression of tollo in the salivary gland interfered with normal morphogenesis. 

Two major points are made. One is the exclusion of a subset of genes expressed in the early stage of salivary gland development. The other is the significance of tollo exclusion in salivary gland development. This reviewer found uncertainty in both claims, which the authors must address. Given the weakness in the exclusion model and some uncertainty in the presentation of the data, this reviewer is not convinced of the magnitude of the progress made in this version of the manuscript. 

Major point. 
1.	Handling and interpretation of single-cell RNA sequencing data. 
1-1	If I understand correctly, the authors obtained scRNAseq data of purified fkh>GFP+ cells and arm-GFP cells and combined the two data sets before clustering. Then, each cluster (Fig. 3A, Fig. 4A) contains cells of fkh>GFP and arm-GFP fractions. Information on the contribution from each FACS sorted fraction would help interpret the data. This will also provide information on the purity of FACS sorting.

*Our rationale behind this experimental approach was the fact that, as pointed out in our manuscript, salivary gland primordium cells only comprise 200 cells per embryo, which in an embryo of at stage 10-11 constitute about 0.4% of all embryonic cells. For this reasons, several past methods to look more globally at expression changes in embryonic cells had failed to identify placodal cells specifically (as discussed in our introduction). Any enrichment of the placodal population with respect to all other embryonic cells would therefore increase our chances of being able to analyse a meaningful number of salivary gland placodal cells and their expression profiles in the early stages of morphogenesis.*

*Our FACS isolation method was not intended to give a clean population of only placodal cells, we never aimed for ‘purity’ but rather enrichment through the FACS process. Our further rationale was that in amongst an enriched population of cells, we could identify placodal cells based on either GFP expression (our genetic label) or based on expression of previously well characterised maker genes such as Scr (at the top of the specification cascade / Henderson, K. D., and D. J. Andrew, 2000 Regulation and function of Scr, exd, and hth in the Drosophila salivary gland. Dev. Biol. 217: 362–374; Zhou, B., A. Bagri and S. K. Beckendorf, 2001 Salivary gland determination in Drosophila: a salivary-speciﬁc, fork head enhancer integrates spatial pattern and allows fork head autoregulation. Dev. Biol. 237: 54–67) or Fkh or Hkb expression (two genes key to morphogenesis in the glands; Sánchez-Corrales, Y. E., Blanchard, G. B. & Röper, K. Correct regionalization of a tissue primordium is essential for coordinated morphogenesis. Elife* ***10****, e72369 (2021).*

*Combination of the salivary gland-marked cell data (fkhGal4 x srcGFP) with epidermis-marked data (ArmadilloYFP) made identification of cell types other than salivary gland in the dataset easier due to large cell numbers upon combination.*

*Analysis of the ‘fkhGal4 x srcGFP’-only data (identified by being GFP+) would lead to a UMAP with fewer cells but identical clusters, see below, and we decided to keep the combined data analysis. We also show below the percentages of cells identified per genotype or cluster for your information.*

*Below is a mock version of Figure 2 using just the srcGFP cells for UMAP generation, and apart from a different shape of UMAP the result is the same.*

1-2	The main claim derived from the analysis shown in Fig. 2 is that the collected cells are classified into five clusters representing the temporal order of salivary gland development (early, specified duct, specified secretory and post specification). I repeat the question of 1-1: To what extent the two FACS sorted cell fractions contribute to each cluster? Some of the none-salivary gland cell clusters (early and specified duct) may include more cells from the arm-GFP fractions, which should be derived from the broad ectodermal territories not covering the PS2. Then how did the author justify their naming?

*We assume the reviewer is referring to Figure 3 and not Figure 2 here, as Figure 3 is the one focussing in on the salivary gland lineage and analysing the 4 clusters (now named ‘progenitor and early salivary gland’, ‘specified duct’, ‘specified secretory’ and ‘post specification/late salivary gland’). The HCR RNA localisation analysis across early stages of tube formation clearly identified the four clusters as matching the descriptive terms given by us.*

*The six clusters identified and named in Figure 2 were determined based on our analysis of marker gene expression pattern, as determined by HCR in situ shown in the figure.*

*As already discussed above, to explain and illustrate in more detail what is known with regards to specification of the salivary gland placode and its known subdivision into future duct and future secretory cells (a large body of studies dating back more than 20 years: Kuo, Y. M. et al. Salivary duct determination in Drosophila: roles of the EGF receptor signalling pathway and the transcription factors fork head and trachealess. Development* ***122****, 1909–1917 (1996)) we have firstly now included schematics in Figure 1A’ and A’’ to explain this, and have also explained this in more detail in the manuscript. We have also added explanatory schematics to the single channel panel composite in Suppl.Fig. S4A-E so that it is easier to appreciate which cells are in fact labelled by the HCR in situ.*

*See also more detail on this above in response to Reviewer 2.*

*The cluster termed ‘early’ in Figure 3 and now termed ‘epidermal and early salivary gland’ in Figure 2 is likely to contain both epidermal cells, epidermal cells about to be fated as salivary gland and early just specified salivary gland cells, as their expression profiles are likely to be similar.*

*Please refer to our further analysis and discussion above in response to the other reviewers.*

*Furthermore, ArmYFP-marked cells are expected to contribute to salivary gland cell clusters as salivary gland cells are epithelial and thus do express armadillo as a key epithelial gene/protein all throughout their morphogenesis and later functional life.*

2.	The strong point made in this manuscript is the identification of some genes excluded from the salivary gland to allow gland development. This class of gene includes grh, hth and tollo/toll8. 

2-1	Regarding the "non-salivary gland epidermal cells " The authors presented tollo expression (Fig. 4E, 6AB), in which the authors described the gene as expressed at the time of salivary gland placode specification (page 11, paragraph 1). I am not convinced with this view of tollo "exclusion". The tollo gene seems simply not expressed in the placode: no solid evidence for active exclusion of tollo expression from the salivary gland placode is presented. "Exclusion" is a strong word. It should be presented with robust evidence. Although the ectopic expression of tollo interfered with the salivary gland development, such an effect has been described in other cases (ex. <https://doi.org/10.1002/dvg.20245>), and its implication is limited.

*Pease see our detailed answer to the points raised here below in response to point 6 that raises the same question.*

*As we set out below and in Figure 6, Toll-8/Tollo is clearly initially expressed in cells that also begin to express fkh, downstream of the salivary gland fate specification cascade, but then the future secretory cells of the placode, but not neighbouring epidermal cells and also not the salivary gland duct-fated cells, downregulate/exclude/lose expression of toll-8/tollo.*

*In the original version of the manuscript we had already identified that the downregulation and exclusion of expression is downstream of the most upstream identity-specifying transcription factor Scr: in Scr-/- mutants the earlier expression of toll-8/tollo in parasegment 2 is maintained. We also already showed that one of the key transcription factors downstream of Scr that is key to the initiation of cell shape changes, Fkh, is not required.*

*A good candidate for the regulation of transcriptional exclusion is the transcriptional repressor Hairy (h) that has previously been implicated in control of salivary gland morphogenesis (Myat, M. M. & Andrew, D. J. Epithelial tube morphology is determined by the polarized growth and delivery of apical membrane. Cell* ***111****, 879–891 (2002)). In fact, the published hairy mutant phenotypes in glands, with aberrant, too wide and/or branched lumens are highly reminiscent of the phenotypes we have observed here with Toll-8/Tollo and Toll-6 overexpression. We therefore have now analysed toll-8/tollo as well as toll-6 expression by HCR in situ in hairy mutants (the allele h[25], using h[25]/TM3 twi::GFP). In these mutant embryos, toll-8/tollo and toll-6 expression is retained in parasegment 2, strongly suggesting that Hairy is upstream of and possibly directly responsible for the downregulation and exclusion of toll-8/tollo and toll-6 expression in the salivary gland placode. These data are now included as Fig. 6E and Suppl. Fig. S7D.*

2-2	Down-regulation of grh and hth was clearly documented (Fig. 4E, S3A). But their functional implication is not known.

*We agree, both grh and hth also show the exclusion/downregulation/loss. As explained in detail below (point 6) and also in the original version of the manuscript, this was known and analysed previously for hth (Henderson, K. D. & Andrew, D. J. Regulation and function of Scr, exd, and hth in the Drosophila salivary gland. Dev Biol* ***217****, 362–374 (2000)), but not for grh.*

*We decided to focus our initial analysis on a morphogenetic effector showing this behaviour rather than a transcription factor, but will certainly analyse the grh exclusion in more detail in future studies.*

Other comment.
3.	Log2 fold change values should be clearly defined. In their second reply to my inquiry, the authors presented a table listing group 1 and 2 cells for the log2 fold change value calculation. The choice of groups 1 and 2 differed from one data set to another. That description was totally absent in the original manuscript and made it impossible to evaluate the data. In the table, I believe "Figure 2C" should be "Figure 3C". Is the description for group 2 cells described as "Remaining cells in the dataset" for the description of Figure 2C (which I believe is Figure 3C) available at any place in the manuscript?

*I repeat our response form above:*

*The method used to generate log2fold change values in this paper was through Seurat’s differential expression functions, either ‘findallmarkers’ or ‘findmarkers’ (a general breakdown of these functions can be found under the heading Finding differentially expressed features (cluster biomarkers) in the introductory tutorial for using Seurat;* [*https://satijalab.org/seurat/articles/pbmc3k_tutorial.html*](https://satijalab.org/seurat/articles/pbmc3k_tutorial.html)*).*

*The values of log2fold in the paper are based on the value for each gene in the avg_log2FC column and using the default Wilcoxon Rank Sum test.*

*We have now added a table to the methods, listing for each figure panel the groups that were compared and this is now also generally explained in the figure legends.*

4.	Page 8. 2nd paragraph. "pip (pipe), a sulfotransferase of the Golgi is key to the later secretion function of the salivary glands and a known marker of these, and its transcript colocalised already early on with fkh mRNA, confirming this cluster as 'salivary gland' (Fig. 2B, B'). "
Misleading logic. This data confirmed that one gene enriched in this cluster, pipe, is expressed in the salivary gland. If the authors intend to claim this cluster as a 'salivary gland', the expression of multiple genes should be tested before making a firm conclusion.

*The identification of pipe and various other factors involved in secretion in the salivary glands was not based on our analysis provided here, but on previous studies by the Andrew lab (Fox, R. M., Hanlon, C. D. & Andrew, D. J. The CrebA/Creb3-like transcription factors are major and direct regulators of secretory capacity. J Cell Biol* ***191****, 479–492 (2010)), hence we do not present a circular argument but base our cluster identification on previously published knowledge.*

*We now make this clearer in the relevant section of the manuscript.*

5.	Page 8. last paragraph. "Thus, the single cell RNA-sequencing analysis of cells marked by and isolated based on GFP expression under fkhGal4 control (UAS-srcGFP fkhGal4) was able to generate a cell atlas of the salivary gland placode as well as its precursor epidermis and nearby tissues at early stages of embryogenesis that provides a rich resource of expression data for these stages. "
This reviewer is not convinced with this argument. The muscle, CNS, hemocyte clusters may be simply a contamination.  Other possibility is that additional genes are activated during cell dissociation and FACS sorting. It was reported that Notch signaling is activated by dissociation-dependent ectodomain shedding of Notch receptor. The E(spl) positive clusters may be such an example.

*Our interpretation, which we are happy to elaborate on in the discussion, is that the other clusters are a ‘contamination’ (co-isolated by FACS), as they are either cells that are in very close contact to the salivary glands or represent cell types that are part of the epidermis pre-salivary gland specification.*

*These cells might also have been included by gating during the FACS process that was not at its most stringent, as our aim was to isolate as many salivary gland placodal cells as possible (due to their very low number), and to then (as we have done) use HCR in situ to confirm cluster identities. We also show now (above for reviewer 1 and in Suppl. Fig. S1) that fkhGal4-driven membrane-GFP (srcGFP) is expressed in a few extra cells in addition to those endogenously expressing fkh, likely because this Gal4 line is based on only a 1kb fragment of the total much larger fkh promoter.*

*Furthermore, our HCR analysis and analysis of GFP-reporters of E(spl) loci identified, as we show in Figure 4D, clearly demonstrates expression of the E(spl) genes in the embryonic epidermis at the time of salivary gland specification, overlapping with future duct-fated cells. Furthermore, we have now included in Suppl. Fig. S5C panels of E(spl)-m4-BFM HCR in situs compared to fkh in situs illustrating very clearly that at gland specification stage (late stage 10) when fkh expression has just initiated at the position of the future invagination point, that E(spl)-m4-BFM is still expressed across parasegment 2, in a pattern similar to the neighbouring parasegments, and that shortly after at the onset of apical constriction at mid stage 11 its expression is now downregulated and specifically excluded from the salivary gland secretory cells.*

6.	Page 11, para1, "At the gland specification stage, both tollo and grh were still expressed in parasegment 2 where the salivary gland placode will form, but both were clearly excluded from the secretory part of the placode once apical constriction commenced. "
(This comment overlaps with comment 2-1). I agree with the authors' view that grh is initially expressed in the salivary gland placode and down-regulated afterward. But I disagree that tollo is expressed in the salivary gland placode (left most image). There is a signal in the placode, but the level seems to be the same range in the later stage. The tollo expression pattern shown in Fig. 6A indicates this gene is expressed in a striped pattern outside of the sg placode. Without additional expression study on the earlier stage with higher tollo expression, the statement of tollo down-regulation is not supported. 
  Is the low tollo expression in the placode is due to the salivary grand program?   The authors provided a data to tollo expression in Scr mutant (Fig. 6D), which showed slight upregulation. Since Scr is involved in a broader program of segment identity, implication of the result should be carefully evaluated. I feel this level of tollo expression change is not sufficiently strong to justify the "exclusion" model. The authors also showed that tollo expression does not change (upregulated) in fkh mutants (Sup Fig. 4C), arguing against the exclusion model.

*I repeat my answer for reviewer 2, point 1) with regards to the toll-8/tollo downregulation:*

*To demonstrate the downregulation and exclusion of toll-8/tollo expression more clearly, we have now quantified it. We have used late stage 10 embryos (gland specification) and embryos at late stage11 to stage 12 (continued invagination) and have measured toll-8/tollo HCR fluorescence intensity per area for the area of the secretory salivary gland placodal cells in parasegment 2 (as identified by fkh expression) and have compared this in each embryo to toll-8/tollo fluorescence intensity per area for an equivalent patch of cells in parasegment 1, where the overall intensity does not change between these stages. Expressing toll-8/tollo intensity as a ratio between PS1/PS2, as this allows to compare between different embryos with variation in HCR fluorescence signal. This quantification revealed a clear loss of fluorescence intensity for HCR for toll-8/tollo in PS2 between late stage 10 and stage 12 (now Fig. 4F’).*

*We also now show the earliest and second-to-last panel of Fig. 3F for tollo expression in Suppl. Fig. S5C as inverted b&w panels, with the position of the placode and parasegment 2 labelled, as this clearly shows the loss of HCR label.*

*With regards to downregulation or exclusion of expression in the placode:*

*The first example of a gene reported to be ‘specifically lost’ from the salivary gland placode was the homeotic factor Homothorax (Hth), shown by Debbie Andrew’s lab in 2000 (Henderson and Andrew, 2000, see full reference in our manuscript), as we discuss in our manuscript in results (page 9) and discussion (page 15). Henderson et al. state “Since hth expression is speciﬁcally lost in the salivary gland primordia (Figs. 3C–3F; Rieckhof et al., 1997; Kurant et al., 1998), we tested if Scr regulates hth expression in the salivary gland primordia. In embryos lacking Scr function, hth mRNA and HTH protein are maintained in the cells that would normally form the salivary gland beyond stage 11 (Figs. 5A and 5C and data not shown).”*

*This same paper in depth analyses the Scr/Hth/Exd relationship in defining salivary gland identity, and the combination of all three factors is required: in individual zygotic mutants for Scr, in individual zygotic mutants for hth and in individual maternal/zygotic mutants for exd no salivary gland specific genes are ever expressed, the ventral region in parasegment 2 looks and behaves like epidermis.*

*With regards to tollo downregulation:*

*1) In Figure 4E, we only show changes to tollo expression by HCR during the relevant stages relating to salivary gland specification and early morphogenesis, i.e. late stage 10 (‘gland specification’), stage 11 (‘pre-apical constriction’ and ‘apical constriction’) and late stage 11/early stage 12 (‘continued invagination’). An even earlier stage, early stage 10, is shown in Figure 6A, a stage when salivary gland placode specification is just taking place and fkh expression is only present in the more posterior and ventral part of the salivary gland placode (a pattern previously published by Myat and Andrew, 2000, Development) before spreading across the whole placode (as analysed in detail with endogenously-tagged Fkh-GFP by us in Sanchez-Corrales et al., 2021, eLife). As our analysis and images in Figure 6 show, at this early specification stage the mRNA expression of fkh clearly overlaps the undulating stripe pattern of toll-8/tollo (also shown as individual channels now in Suppl. Fig.S7A), just as it also overlaps part of the early stage 10 stripe pattern of toll-2/18w and toll-6. As Figure 6B shows, in our view, this pattern already by late stage 10/early stage 11 resolves to one where toll-6 and toll-8 are now not expressed within the domain where fkh is expressed, whereas, as published previously by the Beckendorf lab, toll-2/18w shows expression at the position of the forming invagination point, an expression that then radially spreads across the placode (Kolesnikov, T. & Beckendorf, S. K.. Dev Biol* ***307****, 53–61 (2007)).*

*The early embryonic stripe expression pattern of Tolls up to and including germ band extension has been analysed in detail by others (Paré, A. C. et al. A positional Toll receptor code directs convergent extension in Drosophila. Nature 1–16 (2014) doi:10.1038/nature13953; Patricia L. Graham, W. Ray Anderson, Elizabeth A. Brandt, Jie Xiang, Leslie Pick, Dynamic expression of Drosophila segmental cell surface-encoding genes and their pair-rule regulators, Developmental Biology,Volume 447, Issue 2, 2019, Pages 147-156). Toll striped expression during germ band extension has been shown to be key to direct myosin accumulation in cells that in turn drives cell intercalation pattern that are part of the motor driving germ band extension (work by the Zallen, Lecuit and Sanson labs). We did not investigate Toll expression patterns across all and in particular earliest embryonic stages because the patterns we observed at early stage 10 appeared to match the ones previously published in particular by the Zallen lab:*

*(Paré et al., 2014, Nature)*

*With regards to Scr’s role in the specific loss of expression/downregulation of expression/exclusion of expression of toll-8/tollo, as already cited above we know from extensive work by the Andrew lab that Scr (and also its co-factors hth and exd) is essential for all salivary gland specific expression at earliest stages of salivary gland placode specification.*

*Scr’s function to specify the salivary gland placode is restrained to the actual position of the placode by a variety of other transcription factors and signaling, analysed by the Debbie Andrew (Andrew et al., Setting limits on homeotic gene function: restraint of Sex combs reduced activity by teashirt and other homeotic genes. EMBO J (1994)) and illustrated in the below schematic:*

*Based on this, many factors appearing to be salivary gland-specific or required for morphogenesis or function in the tissue have been analysed in scr-/- mutants to assess if their expression is controlled by this most upstream homeotic factor, examples are:*

*Fkh is lost in Scr-/-: Panzer, S., Weigel, D. & Beckendorf, S. K. Organogenesis in Drosophila melanogaster: embryonic salivary gland determination is controlled by homeotic and dorsoventral patterning genes. Development* ***114****, 49–57 (1992).*

*Expression of the salivary gland restricted control element of the fkh promotor is lost in Scr-/-: Zhou, B., Bagri, A. & Beckendorf, S. K. Salivary Gland Determination in Drosophila: A Salivary-Specific, fork head Enhancer Integrates Spatial Pattern and Allows fork head Autoregulation. Dev Biol* ***237****, 54–67 (2001).*

*CrebA and eyg are lost in Scr-/-: Henderson, K. D. & Andrew, D. J. Regulation and function of Scr, exd, and hth in the Drosophila salivary gland. Dev Biol* ***217****, 362–374 (2000).*

*Trh is lost in Scr-/-: Isaac, D. D. & Andrew, D. J. Tubulogenesis in Drosophila: a requirement for the trachealess gene product. Genes Dev* ***10****, 103–117 (1996).*

*18w/Toll-2 is lost in Scr-/-: Kolesnikov, T. & Beckendorf, S. K. 18 wheeler regulates apical constriction of salivary gland cells via the Rho-GTPase-signaling pathway. Dev Biol* ***307****, 53–61 (2007)*

*With regards to other upstream regulation of toll-8/tollo expression in addition to Scr:*

*Over the years, a complex picture of regulation of expression of factors in the salivary gland has emerged, summarised in different reviews including our own (see image below) that highlight that only a subset of factors downstream of Scr is then controlled by Fkh, therefore it is not at all unusual to find that toll-8/tollo appears to not be regulated by Fkh.*

*(Sidor, C. & Röper, K. Genetic Control of Salivary Gland Tubulogenesis in Drosophila. in vol. 132 125–149 (2016).)*

*As explained above, we have now tested the repressor hairy for a negative regulatory role upstream of toll-8/tollo and have found that in hairy mutants toll-8/tollo is not downregulated/excluded any longer, and the hairy mutant gland phenotypes (published in the paper cited below) phenocopyToll-6 or Toll-8 overexpression (Myat, M. M. & Andrew, D. J. Epithelial tube morphology is determined by the polarized growth and delivery of apical membrane. Cell* ***111****, 879–891 (2002)).*

7.	Staging description should be consistent. The text sometimes followed the staging of Campos-Ortega and Hartenstein, in other place "start of gland specification", "apical constriction", etc are used.

*We apologise if the reviewer found the description of stages confusing. The salivary gland placode becomes specified over stage 10, with fkh expression commencing in a few cell early stage 10, and with any obvious morphogenetic changes only commencing and developing across stage 11.*

*As our interest was focussed on expression patterns influencing the early morphogenetic changes, the term ‘stage 11’ is too wide-ranging and broad to capture the morphological changes we are interested in, hence the additional terminology of ‘gland specification’, ‘pre-apical constriction’, ‘apical constriction’ and ‘continued invagination’. We now apply both the more descriptive morphogenetic and the classical stage labeling together to avoid confusion.*

8.	Typo. Page 7 line 17. 17 genes we highly upregulated   -> 17 genes were highly upregulated

*Thanks, we have changed this.*

9.	Fig. 2B-G. Please specify the stage of embryos.

*We have added this.*
