# Supplementary material for: Single-cell analysis of the early Drosophila salivary gland reveals that morphogenetic control involves both the induction and exclusion of gene expression programs
Source: PLoS Biol. 2025 Apr 21;23(4):e3003133. doi: 10.1371/journal.pbio.3003133 (PMC12043239; doi:10.1371/journal.pbio.3003133)
Supplement: Attachment — Submitted filename: Response to reviewers_revision 2.docx [file pbio.3003133.s023.docx]

*With regards to the reviewers remaining comments:*

Reviewer #1: May and Röper have addressed the various issues raised by this reviewer. Notably, they have made an effort to compare several similar studies and provide a more insightful analysis of Tollo receptors. These revisions have improved the manuscript's clarity. While the results regarding Tollo receptors remain inconclusive, they are potentially interesting. The manuscript, however, remains primarily descriptive to my opinion. 


Reviewer #2: The authors have addressed my concerns, and this version is more clear and easier to follow.

The following two points are suggested edits:

Rebuttal statement: "Furthermore, with regards to potential inter-regulation of Toll expression, we have now analysed toll-2/18w expression and also toll-6 expression when Toll-8/Tollo is overexpressed and find that there is no change to either of the other two Tolls at the transcriptional level (see Suppl. Fig. S6)."

I assume you are referring to Suppl. Fig. S7 instead.

*The reviewer is correct, the citation was incorrect in the rebuttal letter but the supplementary figure is correctly cited in the manuscript.*

Rebuttal statement: "As already explained above, our data (∆cyto expression as well as retained CrebA labelling) demonstrate that Tollo/Toll8 is highly unlikely functioning as a cell fate switch."

My question is not whether Tollo is driving the cell fate switch but rather that it is not surprising to detect the downregulation of certain genes as cells differentiate.

What I find helpful is the Tollo expression shown in Figure S5A, as it confirms that Tollo is expressed in a subset of cells within the cluster related to the salivary gland.
I would also encourage you to show Tollo expression in the subset of cells analyzed in Suppl. Fig. S3.  

*We have added the tollo expression on the UMAP to Suppl.Figure S3 as part of showing top marker gene UMAPs for the clusters identified in B as well (as we do already for C in C’, and tollo is part of this).*

Reviewer #3: The authors have improved the manuscript and figures and have answered all my concerns.


Reviewer #4: Main Comment 
The revised manuscript of May and Roper addressed most of the questions I raised for the previous version. Quantification of toll8 mRNA revealed clear down-regulation in the developing salivary gland placode. Additional analysis of the upstream transcriptional program under the control of Scr and hairy provided further insight into toll8's negative regulation. 

Minor Comment
page 14 line 10 from bottom. : Should  (Suppl. Fig. 4C) be Suppl. Fig. 7C?

*This is correct, thanks for spotting this.*

*With regards to the general comments from PLOS Biology:*

1 - TITLE: We would like to propose a small tweak to the title. If you agree, we suggest that you change it to:

Single-cell analysis of the early Drosophila salivary gland reveals that morphogenesis is regulated by both the induction and exclusion of gene expression programs.

*If possible we would prefer the below compromise version between our original and your suggested title:*

**Single-cell analysis of the early *Drosophila* salivary gland reveals that morphogenetic control involves both the induction and exclusion of gene expression programs**

2 - FINANCIAL DISCLOSURES: Please take a moment to update your financial disclosures statement in our online system. A complete financial disclosures statement will include the following details: 

Initials of the authors who received each award
Grant numbers awarded to each author
The full name of each funder
URL of each funder website
A statement indicating whether the sponsors or funders played any role in the study design, data collection and analysis, decision to publish, or preparation of the manuscript

*”This work was supported by the Medical Research Council, as part of United Kingdom Research and Innovation (also known as UK Research and Innovation; https://www.ukri.org/councils/mrc/) [MRC file reference number MC_UP_1201/11 to KR]. For the purpose of open access, the MRC Laboratory of Molecular Biology has applied a CC BY public copyright licence to any Author Accepted Manuscript version arising. The funders had no role in study design, data collection and analysis, decision to publish, or preparation of the manuscript.”*

*This has been done, confirmed with the journal, will be added in the correct section in the resubmission process.*

3 - DATA: thank you for depositing your RNA-seq data on GEO. I see that this is currently private - which is OK for now, but please note that this will need to be made public before publication.

*This is now public.*

4 - DATA: For figures containing flow cytometry data data, we ask that you provide FCS files and a picture showing the successive plots and gates that were applied to the FCS files to generate the figure. As these files are usually quite big, you can deposit them in the Flow Repository (<http://flowrepository.org/>) and please make sure they are publicly available. 

I have heard that Flow Repository has been giving some AUs issues lately - and if you find that the case, you can use a different repository.

*We attempted flowrepository, but through various browsers and on different devices the website just gets to an error message instead of allowing upload of data.*

*We therefore uploaded the files to figshare, with the following DOI:*

[*https://doi.org/*](https://doi.org/)*10.6084/m9.figshare.28625339*

5 - DATA: In addition to providing the raw RNA-seq and FCS files, detailed above, to be fully compliant with PLOS' Data Policy we also require that all other data be made available without restriction: <http://journals.plos.org/plosbiology/s/data-availability>. For more information, please also see this editorial: <http://dx.doi.org/10.1371/journal.pbio.1001797> 

Note that we do not require all raw data. Rather, we ask that all individual quantitative observations that underlie the data summarized in the figures and results of your paper be made available in one of the following forms:

a. Supplementary files (e.g., excel). Please ensure that all data files are uploaded as 'Supporting Information' and are invariably referred to (in the manuscript, figure legends, and the Description field when uploading your files) using the following format verbatim: S1 Data, S2 Data, etc. Multiple panels of a single or even several figures can be included as multiple sheets in one excel file that is saved using exactly the following convention: S1_Data.xlsx (using an underscore).

*We have added Supporting information files for the raw and analysed data for the following figures:*

*Raw data for plots presented in figures are contained in the following files:*

*Figure 4F’:	‘S1_data.xls’ (raw ImageJ quantification data of fluorescence intensity)*

*‘S2_data.pzfx’ (GraphPad Prism 9 file of fluorsencence intensity ratio data*

*derived from ‘S1_data’ analysed)*

*Figure 5D’:	‘S3_data.xls’ (raw data of apical area quantification)*

*‘S4_data.pzfx’ (GraphPad Prism 9 file of cumulative analysis of apical area*

*quantification data from ‘S3_data.xls’ in size bins)*

*Figure 5E’:	‘S5_data.xls’ (raw ImageJ quantification of fluorescence intensity)*

*‘S6_data.pzfx’ (GraphPad Prism 9 file of fluorescence intensity ratio data*

*derived from ‘S5_data’ analysed)*

*They are cited where necessary in the manuscript and listed in the data availability section, and legends are provided in the manuscript.*

Regardless of the method selected, please ensure that you provide the individual numerical values that underlie the summary data displayed in the following figure panels as they are essential for readers to assess your analysis and to reproduce it:

Fig 5D-E 

NOTE: the numerical data provided should include all replicates AND the way in which the plotted mean and errors were derived (it should not present only the mean/average values).

Please also ensure that figure legends in your manuscript include information on where the underlying data can be found, and ensure your supplemental data file/s has a legend.

Please ensure that your Data Statement in the submission system accurately describes where your data can be found.

6 - CODE:  Thank you for providing the scripts used here on Github.  Per journal policy, for sharing custom code during the course of this investigation, we cannot accept sole deposition of code in GitHub, as this could be changed after publication. However, you can archive this version of your publicly available GitHub code to Zenodo. Once you do this, it will generate a DOI number, which you will need to provide in the Data Accessibility Statement (you are welcome to also provide the GitHub access information). See the process for doing this here: <https://docs.github.com/en/repositories/archiving-a-github-repository/referencing-and-citing-content>

*The code has now been archived via Zenodo and the DOI is:*

*DOI: 10.5281/zenodo.15011856*

*This is also now cited in the data availability section.*
